# FRAME: fast reference-based ancestry makeup estimation tool

**DOI:** 10.1093/bioadv/vbag006

**Published:** 2026-01-12

**Authors:** Pramesh Shakya, Ardalan Naseri, Degui Zhi, Shaojie Zhang

**Affiliations:** Department of Computer Science, University of Central Florida, Orlando, FL 32816, United States; McWilliams School of Biomedical Informatics, The University of Texas Health Science Center at Houston, Houston, TX 77030, United States; McWilliams School of Biomedical Informatics, The University of Texas Health Science Center at Houston, Houston, TX 77030, United States; Department of Computer Science, University of Central Florida, Orlando, FL 32816, United States

## Abstract

**Motivation:**

The availability of large-scale genetic data presents a unique opportunity to study the genetic ancestries of individuals, which requires an efficient and scalable method. The existing global ancestry methods are accurate, but they cannot scale to large genetic datasets. Identity-by-descent (IBD) segments are DNA segments shared by individuals such that they are inherited from a common recent ancestor without recombination. These IBD segments, which reflect co-ancestry, provide an efficient alternative for inferring genetic ancestry.

**Results:**

We introduced a reference-based global ancestry inference method called FRAME (Fast Reference-based Ancestry Makeup Estimation). FRAME utilizes partial local ancestry information estimated through IBD segments. Instead of using sophisticated local ancestry inference methods designed to make the best calls at each site, we employed an efficient IBD method for faster and space-efficient algorithms that are robust to genotyping errors. Additionally, we introduced a new method of panel refinement that can enrich the ancestral homogeneity of individual haplotypes in the reference panel, thus leading to more accurate ancestry composition estimates. We benchmarked the performance of our method with real and simulated data. FRAME consumes ∼10–100 times less memory while maintaining a comparable accuracy.

**Availability and implementation:**

Source code is available at https://github.com/ucfcbb/FRAME.

## Introduction

The ancestral composition of an individual is of interest for research and recreational purposes. With the availability of genome-wide genotype testing, it is possible to estimate the ancestral composition through the study of DNA, the ultimate source of genetic information. In this work, we investigate genetic ancestry, which is the ancestry information inferred solely from an individual’s genetic data. Inference of the ancestral composition of the individuals in a population has a wide range of applications. It helps identify an individual’s ancestral origin (Royal *et al.* 2010) and is also useful for studying disease susceptibility (Mersha 2015; Gautam and Mersha 2023), population structure (Padhukasahasram 2014) and plays an important role in personalized medicine (Lu *et al.* 2014; Ortega and Meyers 2014; Bentley *et al.* 2020). Moreover, it can be used in forensic investigations to trace geographic origins and is also useful for correcting population stratification in genome-wide association studies (Pritchard and Donnelly 2001). In an increasingly globalized world, most people have varying degrees of admixture. While it has been accepted to assign individuals of unknown ancestry to a single population in the past, this approach has been shown to be inaccurate, even when ancestry labels are self-reported (Mathieson and Scally 2020). With this in mind, we investigate the problem of reference-based global ancestry inference (GAI) using local ancestry information in this work.

GAI of an individual is the estimation of the proportion of ancestry contributed by *K* distinct ancestral populations, whereas local ancestry inference (LAI) refers to the process of identifying the ancestral origin of each segment or variant site. Many tools that infer global ancestry exist. Traditionally, principal component analysis has been used to study population structure and global ancestry (Patterson *et al.* 2006). ADMIXTURE is a popular model-based tool that estimates the probability of the observed genotypes using ancestry proportions and population allele frequencies (Alexander *et al.* 2009). iAdmix is also a model-based tool that uses reference population allele frequency and maximum likelihood methods to estimate global ancestry proportions (Bansal and Libiger 2015). It works on both genotype array and sequencing data. In principle, one could call the local ancestry for an individual and aggregate it over the genome to estimate the global ancestry. However, the accuracy of LAI can be compromised at individual variant sites, particularly when the assignment is limited to a single ancestry. Moreover, local ancestry calls are more sensitive to regions with low mappability and high rates of recombination. This, in turn, impacts the global ancestry inferred. Additionally, the user might only be interested in global ancestry in which case, calling local ancestry could be computationally costly. While the existing tools are accurate, they do not scale well to large datasets.

Given the increasing availability of large-scale biobank data, there is a pressing need for scalable methods to address these challenges. Recently, methods for identifying identity-by-descent (IBD) segments are booming and it has been discovered that abundant IBD information is available in large data sets. This is specially true for populations that have been through a recent bottleneck event as the IBD sharing among the individuals is higher (Naseri *et al.* 2021). Since IBD segments are supposedly indicating co-ancestry, such segments provide evidence of ancestral populations’ contribution at the sites covered by the segments and capture signals of ancestry informative markers (Sticca *et al.* 2021).

In this work, we present an efficient IBD-based method for estimating the ancestral composition of a sample using a reference panel, since IBD sharing between groups of the same ancestry is expected to be high. Additionally, the process of inferring IBD segments (or long haplotype matches) can be carried out independently of the number of individuals in the reference panel. With an increasing number of available individuals in the reference panel, the ancestry composition inference will be more reliable, while the time complexity will not be impacted. Our approach leverages long haplotype matches between the query and reference panel and includes a method for refining the reference panel. We evaluate our method’s performance using simulated data. As IBD sharing between population groups can vary in diverse cohorts, an IBD-based method is most effective in populations with high levels of expected IBD sharing. We show this by simulating a population that has been through a recent bottleneck with high levels of IBD sharing. We also apply our methods to the 1000 Genomes Project (1KGP) (The 1000 Genomes Project Consortium *et al.* 2015) and Human Genome Diversity Project (HGDP) data (Bergström *et al.* 2020).

## Methods

### Overview

**Figure 1:**
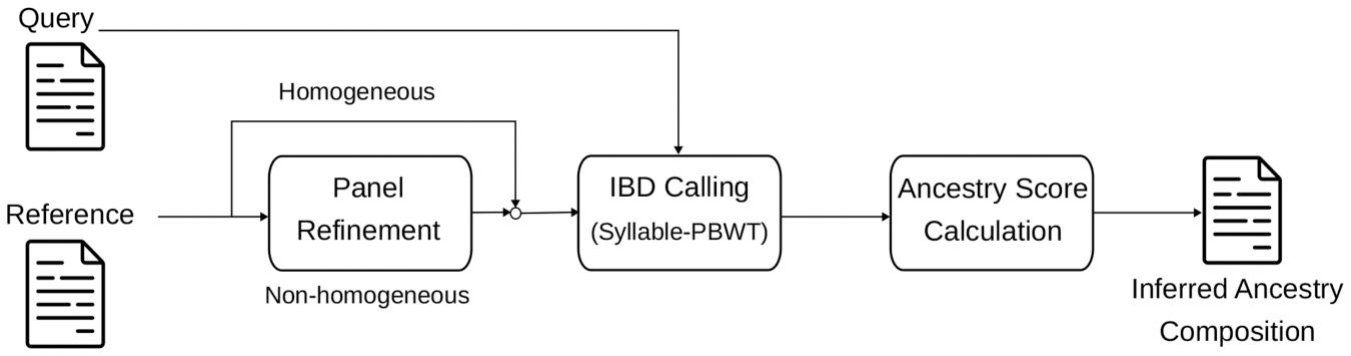
FRAME pipeline. FRAME takes as an input a query panel and a reference panel and infers the genetic ancestry composition of samples in the query panel. The inputs in the form of a reference and query panel are used by the LAI engine to call IBD segments. The input reference panel can also be refined if it consists of non-homogeneous samples.

In this section, we describe the overall working of our proposed method. FRAME is an IBD-based method that uses a reference panel. Figure 1 shows the different components of our method. FRAME takes as input a reference panel and a query panel, both in the form of VCF files. We use Syllable-positional Burrows-Wheeler transform (Syllable-PBWT) (Wang *et al.* 2023), by default, to call an IBD segment between the query panel and reference panel. The Positional Burrows-Wheeler Transform (PBWT) is an efficient data structure introduced by Durbin (Durbin 2014) that facilitates haplotype matching and queries. Syllable-PBWT is a space-efficient PBWT variant that supports finding *L*-long matches between a query haplotype and a reference panel in compressed space. Syllable-PBWT achieves space compression by dividing the input haplotype panel into syllables and building the PBWT on the compressed panel of syllables. Note that in this paper, we use IBD segments to refer to exact haplotype matches. It should be noted that while Syllable-PBWT is used by default, any available IBD-query methods can also be used.

The called IBD segments are processed to assign scores at every site covered by the matching segments. The scores are normalized to account for uneven ancestral population sizes in the reference panel, and the normalized scores are aggregated into ancestry proportions. Figure 1 also shows panel refinement, which can be used to refine the input reference panel to better approximate the source populations. The user has the option to use the panel as is if it consists of homogeneous samples or can choose to refine the panel. The refined panel can be used to improve the accuracy of the inferred estimates.

We define a haplotype *X* as an ordered set of alleles, $\left(x_{0},x_{1},\ldots ,x_{N-1}\right)$, consisting of *N* variants. P is the reference panel consisting of a set of *M* haplotypes $\left(P^{0},P^{1},\ldots ,P^{M-1}\right)$ and Q is the query panel consisting of a set of |Q| query haplotypes, i.e. $\left(Q^{0},Q^{1},\ldots ,Q^{|Q|-1}\right)$. A match between a query haplotype $Q^{i}$ and a reference hapotype $P^{j}$ is defined as $M(Q^{i},P^{j},s,e)$ where the alleles match in range [s,e) i.e. $q_{k}=p_{k}\forall k\in [s,e)$, 0≤s<e≤N. We define $M(Q^{i},P,L)$ as the set of all possible matches that exist between the query haplotype $Q^{i}$ and the reference panel and the matches are at least length *L*. Therefore, $M(Q^{i},P,L)=\overset{|P|-1}{\underset{j=0}{\cup }}\{M(Q^{i},P^{j},s,e)\;|\;q_{k}^{i}=p_{k}^{j}\;\forall k\in [s,e),e-s\geq L\}$. We also define R as an ordered list of unique reference populations and *K* is the number of unique populations, i.e. K=|R|. $r_{k}$ is one such reference population, $r_{k}\in R$ for 0≤k<K. The function $L(P^{j})$ gives the population index of haplotype $P^{j}$ where $L(P^{j})=i$ and $r_{i}$ is the population label of $P^{j}$, $r_{i}\in R$.

### Panel refinement

Admixture is the result of gene flow between two or more divergent or isolated populations. Many genetic ancestry inference methods utilize a reference panel consisting of samples that are representative of possible source populations. Ideally, the chosen panel should consist of haplotypes from the source populations. However, in practice, we often only have access to proxy panels that approximate the source populations. This, in turn, affects the accuracy of the genetic ancestry estimate depending on the quality of the panel used. In this section, we outline our proposed method for refining the reference panel. We refine a panel by increasing the homogeneity of the samples in the panel. We do this by creating synthetic haplotypes. Synthetic haplotypes are created as a mosaic of the segments from existing reference individuals, ensuring that the admixture in the resulting haplotype is more homogeneous and thus better approximates the source populations. A refined panel consists of synthetic haplotypes; hence, we first describe the process to create a synthetic haplotype.

The first step in creating a synthetic haplotype is to specify the minimum admixture proportion for the synthetic haplotype and the target population it should belong to. For instance, we can specify the haplotype to belong to the European population such that the European ancestral contribution should be at least 60%. The next step is to obtain a site-wise ancestry dosage for the existing reference samples that belong to the target population. Site-wise ancestry dosage is the ancestral contribution from each reference population at a site. The site-wise ancestry dosage can be obtained using available local ancestry tools. The initial empty synthetic haplotype with *N* sites is divided into N/W non-overlapping windows of *W* sites each. For each window, we randomly sample a contributor from a pool of candidate haplotypes. The candidate haplotypes are existing reference haplotypes of the target ancestry that satisfy the minimum ancestral contribution cutoff for that window. If candidate haplotypes are not found for a window, we randomly sample from the reference haplotypes. This process is iterated for each window. Since the sampled candidate haplotypes contribute the minimum contribution at each window, the synthetic haplotype created will be homogenous with desired admixture contribution from the target population except for cases when candidate haplotypes are not found for most of the windows.

**Figure 2:**
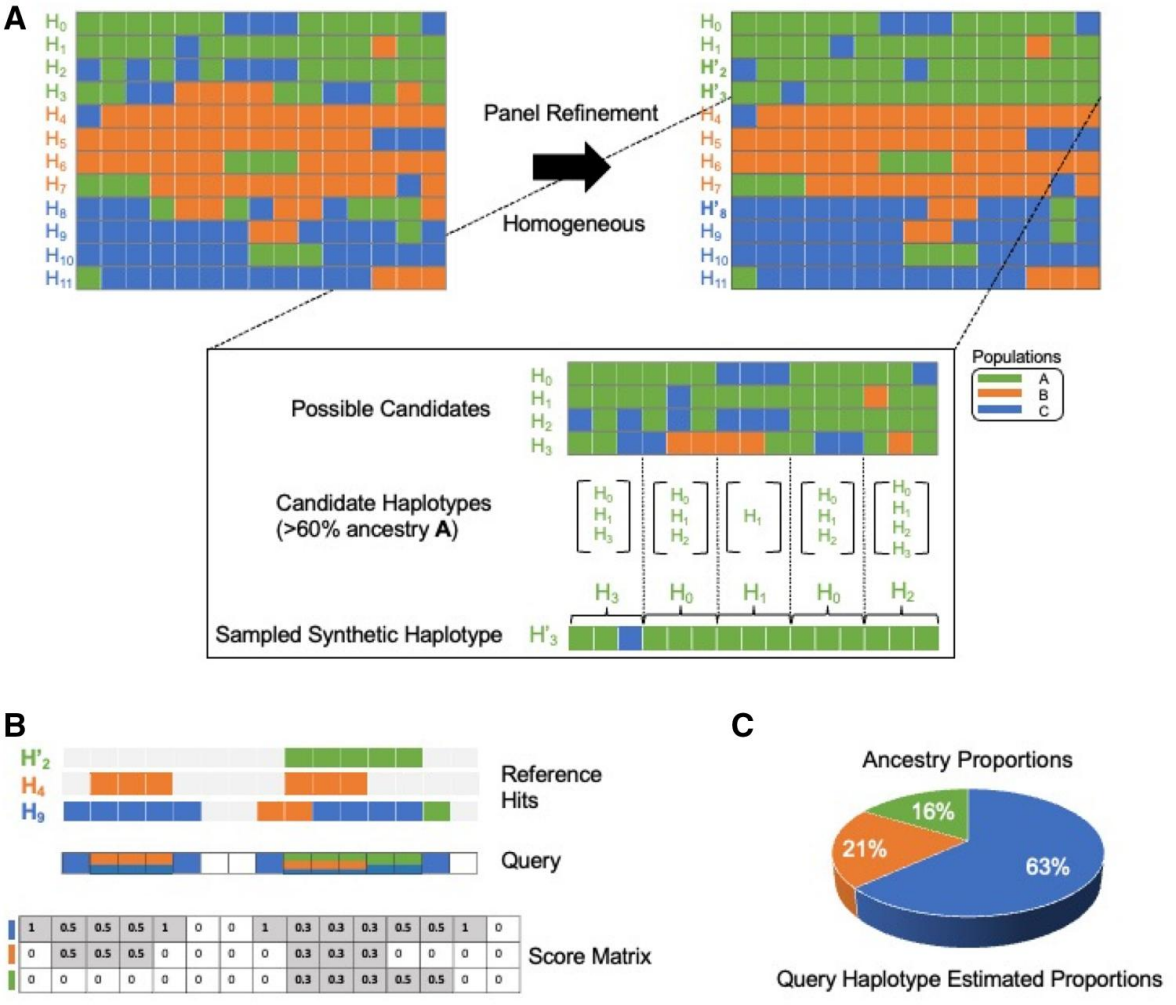
Genetic ancestry composition inference components. (A) Panel refinement. The haplotypes that are ambiguously admixed are replaced with more homogeneous synthetic haplotypes. Homogenous synthetic haplotype $H_{3}^{{\;}^{\prime }}$ that is at least 60% ancestral population *A* is created by sampling segments that are three sites long from $H_{0}$, $H_{1}$ and $H_{3}$ haplotypes of population *A*. The refined panel consists of homogeneous admixed haplotypes such as $H_{2}^{{\;}^{\prime }}$, $H_{3}^{{\;}^{\prime }}$, $H_{8}^{{\;}^{\prime }}$. (B) Site-wise ancestry dosage assignment at sites covered by the IBD matches between a query haplotype and refined panel. The query haplotype is colored at each site to show the site-wise ancestry dosage along with the corresponding score matrix (C) Scores converted into estimated ancestry proportions for the query haplotype.

Figure 2A shows the process of refining the reference panel (left) to obtain a more homogenous panel (right). The original panel consists of 12 haplotypes and 15 sites per haplotype. Haplotypes $H_{0}$ to $H_{11}$ belong to three ancestral populations *A*, *B*, and *C* denoted by the green, orange, and blue colors, respectively, R=(A,B,C). $H_{0}$-$H_{3}$ belong to population *A*, $H_{4}-H_{7}$ belong to population *B* and $H_{8}-H_{11}$ belong to population *C* as denoted by the color of the haplotype labels. Note that the individual cells show the local ancestral population for each haplotype. For instance, even though $H_{3}$ belongs to population *A*, it has local ancestral contributions from populations *B* and *C* as well. Figure 2A shows the process to create a synthetic haplotype $H_{3}^{{\;}^{\prime }}$ of target population *A* such that haplotype $H_{3}^{{\;}^{\prime }}$ consists of at least 60% ancestral contribution from population *A*. The synthetic haplotype $H_{3}^{{\;}^{\prime }}$ is first divided into five windows consisting of three sites each. The zoomed-in panel shows the detailed process of sampling from the candidate haplotypes for each window. The first step is to retain candidate haplotypes for each window from all the possible candidate haplotypes based on our ancestral contribution cutoff. Figure 2A (middle) shows that for the first window, we have $H_{0}$, $H_{1}$, and $H_{3}$ as candidate haplotypes, as these haplotypes constitute at least 60% contribution from population *A* in this window. Similarly, only $H_{1}$ qualifies as a candidate haplotype for the third window. Once we obtain the candidate haplotypes, a random candidate haplotype is chosen for each window. For instance, the first window is sampled from $H_{3}$, the second from $H_{0}$, and so on. The resulting synthetic haplotype is homogeneous and at least 60% of *A* ancestry. The time complexity to create a single synthetic haplotype of target ancestry $r_{k}\in R$ is $O(Ns_{k})$ where $s_{k}$ is the number of samples in the population $r_{k}$.

### IBD calling

In this section, we describe the process of calling the IBDs. Here, we assign site-wise ancestry dosage at each site covered by the IBD segments and aggregate them across the genome to infer the ancestral composition of the query sample. The use of IBD segments provides a fast way to estimate the composition; however, it could result in a lower quality call in some regions compared to local ancestry calls, depending on the coverage of the genome.

We use Syllable-PBWT (Wang *et al.* 2023) to find IBD segments that are at least L sites long. We chose Syllable-PBWT for its efficient compression of the reference panel which inturn improves the compute time to call the IBD matches. The time complexity to precompute the compressed reference panel is O(MN+Mnβ log M) where *n* is the number of syllables and β is a constant factor much smaller than the syllable size. The time complexity to call the IBDs for a single query is $O(N+n\;\text{log}\;M\;\text{log}\;n+n\beta \;\text{log}\;M+\beta c^{\prime }+c^{\prime }\;\text{log}\;n)$ where $c^{\prime }$ is the number of potential matches. However, other IBD query methods (Sanaullah *et al.* 2021; Wei *et al.* 2023) that are recently becoming available can also be used. Once all the matching segments between query samples and the reference panel are found, each site covered by the matching segments will enable the transfer of ancestry labels from the reference panel haplotype to the query haplotype. The final ancestry composition is determined by a scoring process that counts ancestry labels across all sites.

### Ancestry score calculation

The ancestry composition is determined by first estimating the site-wise ancestry dosage. Site-wise ancestry dosage is the ancestral contribution from each reference population at each site covered by the IBD segments. We estimate this by first processing all the IBD segments, followed by a scoring process that counts ancestry labels across all sites. If a site is covered by panel haplotypes from multiple ancestries, their votes are split. The computation of scores is done per query haplotype. Here, we explain the process of assigning scores for one query haplotype for a single chromosome.
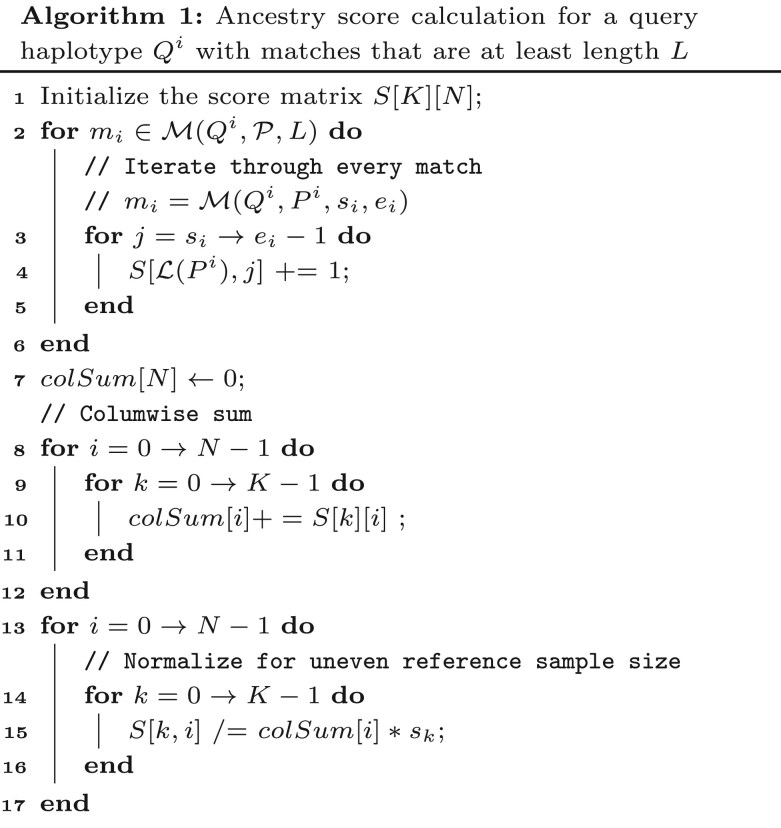


We define a score matrix, *S*, of dimension *K* x *N*, where *K* is the total number of distinct reference populations and *N* is the total number of variant sites in the chromosome. S[i,j] represents a cell in the *i*th row and *j*th column for 0≤i<K and 0≤j<N. S[i,0:N−1] represents the *i*th row from column 0 to column N−1. Each row of the matrix represents one of the reference populations in R, while each column in the matrix represents a variant site on a given chromosome. We iterate through all the IBD segments for a given query haplotype and update the sites covered by each IBD segment. The score matrix counts the number of hits for each site against a reference population. Based on the population of the reference hit, the score for that population is updated across all the sites covered by the matching segment. Algorithm Ancestry score calculation shows the details of processing all the matches, $M(Q^{i},P,L)$ for a query haplotype $Q^{i}$. The time complexity to process the matches and compute the scores for a single query haplotype is $O(N(K+|M(Q^{i},P,L)|))$, where $\left|M(Q^{i},P,L)\right|$ is the number of matches for query haplotype $Q^{i}$. Hence, the total time of score computation for all the matches for all query haplotypes would be O(N(K+c)), where *c* is the total number of matches found between the query panel and the reference panel.

For an IBD segment between a query haplotype and a reference haplotype, say $r_{k}$ is the population that the reference haplotype belongs to. If the IBD extends in the range [i,j) for 0≤i<j≤N, the score matrix S[k,i:j−1] is updated by 1. Figure 2B shows an instance where the query haplotype has five matches with three reference samples, $H_{2}^{{\;}^{\prime }}$, $H_{4}$ and $H_{9}$. The match between the query haplotype and the reference haplotype $H_{9}$ spans in the range [0, 5). Here, R={A,B,C} represents the three ancestral populations and $H_{9}$ belongs to the reference population *A*, i.e. $r_{0}=A$. It should be noted that $H_{9}$ belongs to population *A* as denoted by the color of the label $H_{9}$ even though some of the sites (sites 7,8, etc.) might have a local ancestry corresponding to different populations. The score matrix S[0,0:4] is updated by 1 corresponding to this hit. All the reference hit counts are updated in the score matrix in a similar fashion. After all the matches are processed, the score in each site is weighted by the total column-wise score for that site, i.e. $S[k,i]=\frac{S[k,i]}{\sum_{k}S[k,i]}$ for 0≤i<N,0≤k<K. The weighted scores for each reference population are divided by the number of samples in the reference population to account for sample size bias in the reference panel. The weighted scores for a given haplotype are iteratively calculated for all the autosomes. The weighted score for each reference population is summed across all the autosomes and normalized by the total number of variant sites across all the autosomes. Finally, the normalized scores are converted to ancestry proportions for each individual by dividing the normalized score for each reference population by the sum of the normalized scores across all reference populations for a given individual. Hence, the overall time complexity of inferring the ancestry estimate is the total time required to call the IBDs using Syllable-PBWT and the time spent calculating the ancestry scores. This is sublinear in the size of the reference panel. The space complexity of FRAME is dominated by the size of the compressed reference panel, which is linear to the size of the panel.

## Results

### Data

#### Simulated data

We simulated three datasets to assess the performance of our method. The first two datasets simulate a population that has been through a recent bottleneck event and the third dataset simulates the American admixture model. For the first two datasets, we used SLiM (Haller and Messer 2019) to simulate chromosome 20 for 20 generations with the mutation rate of $10^{-8}$ per base pair per generation. For the third dataset, we used msprime (Baumdicker *et al.* 2022) as it has a well-defined American admixture demographic model. We used the deCode map (Halldorsson *et al.* 2019) for the recombination rates across all datasets. We introduced genotyping errors at various rates (*g* = 0%, 0.05%, 0.1%) in the simulated data sets to test the robustness of the method.

The first dataset simulated a 3-way admixed population using samples from 1KGP data as our source populations. We used 99 samples from Utah residents with Northern and Western European ancestry (CEU), 103 samples from Han Chinese in Beijing, China (CHB), and 108 samples from Yoruba in Ibadan, Nigeria (YRI) as the source populations. We added a bottleneck in the second generation, restricting only 70 individuals from each source population, and grew the population size back to 167 for YRI, 167 for CEU, and 166 for CHB. The admixture was introduced in the third generation, where the admixture probability was evenly distributed across the source populations. The admixed population grew exponentially from the sixth generation to the 18th generation at the rate of 1.73 per generation. In the last generation, 500 individuals were sampled from a population of 70 000 admixed individuals. A tree sequence file and a VCF file were generated as output. The VCF panel consisted of 500 randomly sampled individuals and 11 926 variants after filtering variants with minor allele frequency (MAF) <0.05. The panel was divided into a query panel of randomly sampled 100 individuals and a reference panel of 400 individuals.

The second dataset simulated four sets of 2-way admixed populations using 99 CEU and 108 YRI samples as the source populations with varying admixture proportions. The four sets consist of admixed populations with 55:45, 60:40, 70:30, and 80:20 admixture in both directions of source populations. The simulation setup follows the 3-way simulation until the third generation. In the third generation, we create two admixed populations. For each set, the first population is CEU-biased and the second is YRI-biased. For instance, for the 70:30 set, the first population is at least 70% CEU, whereas the second population is at least 70% YRI. These admixed populations grew exponentially from the sixth generation to the 18th generation at the rate of 1.73 per generation. In the final generation, a total of 3000 samples were selected, with half of the samples being YRI-biased and the other half being CEU-biased. We obtained the true local ancestry values and calculated the admixture proportions for these 3000 samples from the output tree sequence. Next, a total of 100 query samples are randomly selected, with half coming from the first population and the other half from the second population. Similarly, 400 reference samples are also randomly sampled such that there is no overlap with the query samples, and half of the reference samples are YRI-biased, and the other half is CEU-biased.

For the third dataset, we simulated chromosome 20 to closely replicate the AmericanAdmixture_4B11 (Gravel *et al.* 2011; Browning *et al.* 2018) model specified in stdpopsim (Adrion *et al.* 2020), where the source populations consist of European (EUR), African (AFR), and Asian ancestry. We used the deCode map for recombination rates and used the default simulation parameters. We sampled 1000 individuals from the admixed population and 250 individuals each from the EUR, AFR, and Asian source populations.

For all three data sets, we used tskit (Baumdicker *et al.* 2022) to process the output tree sequences to obtain true local ancestry values. These local ancestry values were then aggregated for every individual to calculate the true global ancestry proportions.

#### 1000 genomes Project data

We utilize the 1KGP (The 1000 Genomes Project Consortium *et al.* 2015) data, which was lifted to the hg38 build, consisting of 2504 individuals, to benchmark the accuracy and runtime of our tool for ancestry estimation. The samples in this data are categorized into five self-reported continental labels: European (EUR), African (AFR), American (AMR), East Asian (EAS), and South Asian (SAS). We retain 6 859 490 SNP sites after filtering variants with an MAF <0.05, multiallelic sites, and any insertion/deletions across all the autosomes. To assess the estimated ancestry proportions, each chromosomal panel was split into a reference panel and a query panel. The query panel consists of 500 randomly sampled individuals, and the reference panel comprises the remaining 2004 individuals.

We also benchmark our tool using the Human Genome Diversity Project (HGDP) data (Bergström *et al.* 2020) as a reference panel to show the effect on estimated ancestry proportions. For this, we downloaded the VCF panels that consisted of 802 samples. These samples belong to seven regions: Europe (EUR), Central/South Asia (CSA), America (AMR), Africa (AFR), Middle East (MEA), East Asia (EAS), and Oceania (OCA). For our experiments, we excluded samples from the Middle East and Oceania regions. The panels were also filtered to retain bi-allelic SNP sites with no missing alleles. This filtered panel was phased using SHAPEIT5 (Hofmeister *et al.* 2023) with deCode map (Halldorsson *et al.* 2019) and default parameters. The phased panel was intersected with the previously subsampled 1KGP query panel to obtain a new set of query panel and reference panel consisting of 557 972 variants across all autosomes.

### Benchmarked tools

We compare our estimated genetic ancestry composition with other state-of-the-art tools. Here, we compare the results of FRAME with FLARE (v.0.3.0) (Browning *et al.* 2023), RFMix (v2) (Maples *et al.* 2013), Gnomix (Hilmarsson *et al.* 2021), iAdmix (Bansal and Libiger 2015), ADMIXTURE (Alexander *et al.* 2009), and Recomb-Mix (Wei *et al.* 2025). FLARE, Gnomix, and Recomb-Mix are newer tools that compute local ancestries, whereas RFMix is a model-based tool that is traditionally used for LAI. ADMIXTURE and iAdmix are also model-based tools traditionally used for inferring global ancestry. For the outputs from the local ancestry tools, we aggregate the local ancestry calls over an individual to obtain the ancestry proportions. We use the same genotype data, genetic map, and reference panel for all the tools. All tools were used with default values for their statistical parameters. FLARE was run with posterior probabilities turned on (probs=True) and off. ADMIXTURE was run in the “supervised” mode, where a single input panel contains the reference individuals and the query samples. Table S13 (see online supplementary material) provides detailed commands used to run all the tools. We also aggregated the site-wise ancestry dosages (for tools that output such calls) for each individual across the genome. Such aggregated estimates for a tool are represented by the tool names superscripted with the “+” symbol.

### Evaluation on simulated data

We benchmark the accuracy of FRAME’s estimated admixture proportions using 3-way and 2-way admixed simulated data. IBD segments that are at least 500 sites were used to compute the estimated proportions. We measure the accuracy using two metrics: root mean squared error (RMSE) and Kullback–Leibler (KL) divergence. We calculate the RMSE per individual by comparing the estimated ancestry proportions with respect to the true ancestry proportions using the formula: $\mathrm{RMSE}(\tilde{p},p)=\sqrt{\frac{1}{k}\sum_{i=1}^{k}{\left({\tilde{p}}_{i}-p_{i}\right)}^{2}}$. Here, $\tilde{p}$ is the estimated ancestry proportion for a query individual, *p* is the true ancestry proportion, and *k* is the number of ancestral populations. KL divergence is calculated similarly using the formula: $D_{KL}(\tilde{p}||p)=\sum_{i=1}^{k}p_{i}\;\text{log}\;\frac{\tilde{p}i}{p_{i}}$. For both metrics lower values indicate more accurate estimates. Additionally, we demonstrate the improvement in accuracy achieved by using site-wise ancestry dosages to calculate ancestry proportions and by utilizing the refined panel.

**Figure 3:**
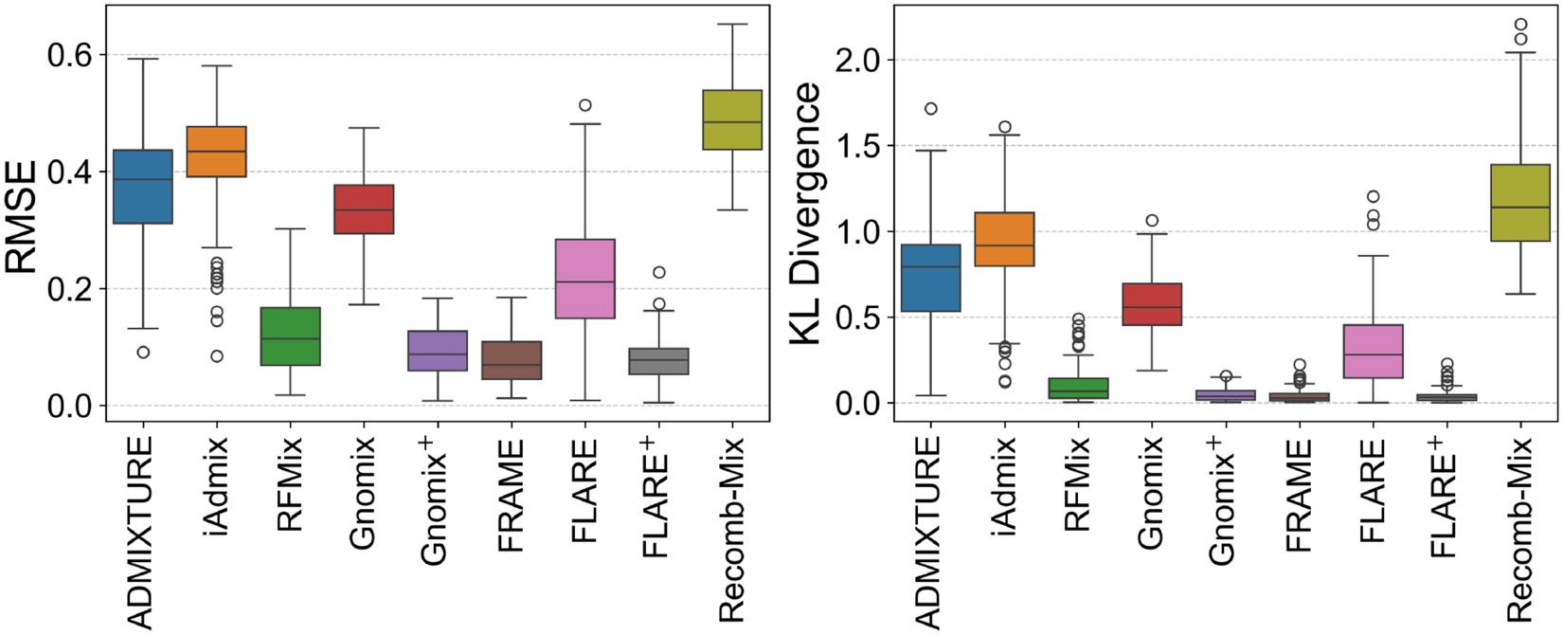
Accuracy comparison for 3-way admixed simulated data (*g* = 0.05%).

FRAME shows robust performance as it has the most accurate estimate of the admixture proportions for the 3-way admixed data shown by the smallest mean RMSE and KL divergence values for different genotyping error rates. Figure 3 shows both RMSE and KL divergence values for 3-way admixed data at 0.05% genotyping error. We observe that FRAME has the lowest mean RMSE (0.0763) and the lowest mean KL divergence value (0.0363) for this dataset. This remains true for 3-way admixed data without any genotyping error and with genotyping error at 0.1% (Fig. S1 and Table S1—see online supplementary material). FRAME has the lowest mean and median KL divergence values compared to all other tools. We ran Recomb-Mix with an MAF filter (f = 0.1), which improved its accuracy. This is shown by Recomb-Mix-f in Fig. S1 and Tables S1 and S2 (see online supplementary material).

**Figure 4:**
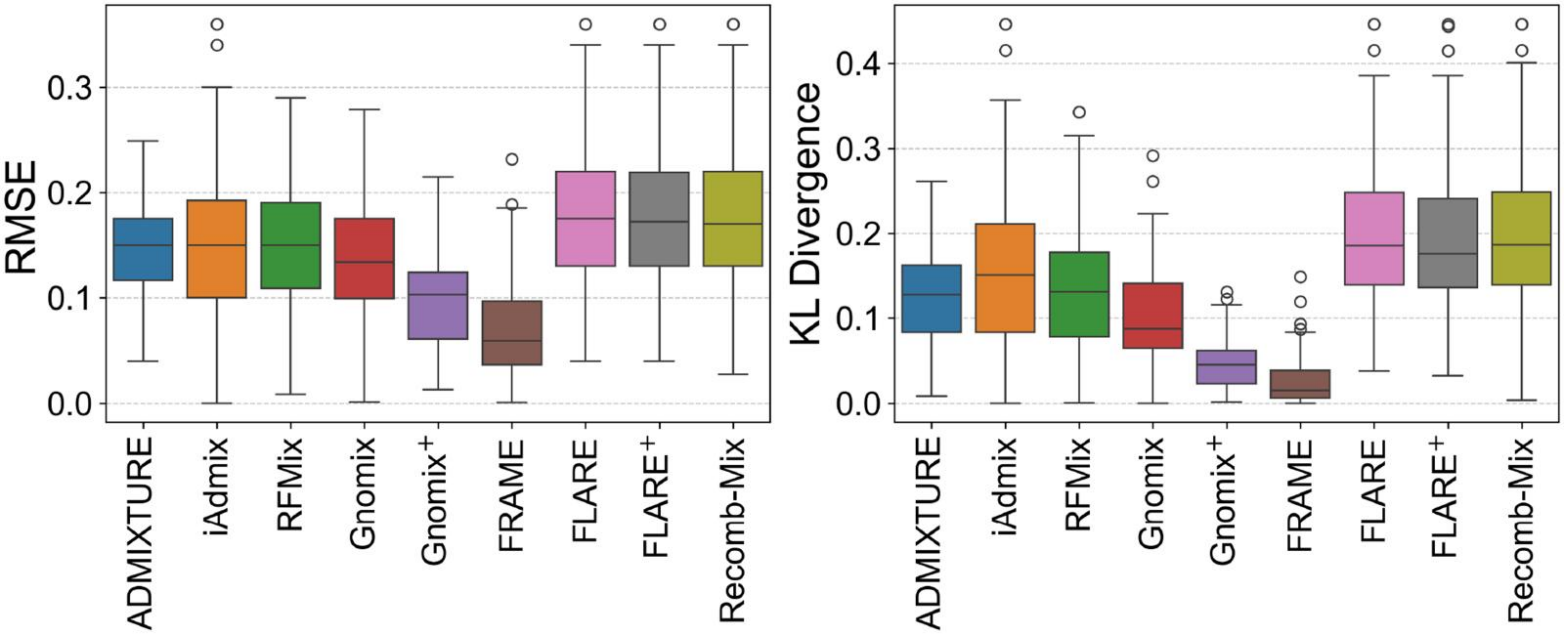
Accuracy comparison for 2-way (80:20) admixed simulated data (*g* = 0.05%).

We observe similar results for 2-way admixed data when running the tools with default parameters. Figure 4 shows that FRAME has the lowest mean RMSE (0.0725) and KL divergence value (0.0265) for 80:20 admixed set at *g* = 0.05% (Tables S3 and S4—see online supplementary material). This result is also true for the 70:30 admixed set (Fig. S3 and Tables S5 and S6—see online supplementary material). However, for the 60:40 and 55:45 admixed sets, ADMIXTURE has the most accurate estimate (Tables S7–S10—see online supplementary material). For the 60:40 and 55:45 sets, the reference panel consists of ambiguously admixed individuals, i.e. evenly distributed admixture proportions. This affects the tool’s accuracy in estimating the proportions. Note that Recomb-Mix has the best accuracy for all variations of this dataset with the MAF filter (Figs S2–S5—see online supplementary material for a colour version of these figures). For 2-way admixed data, such allele frequency filtering is expected to improve the result; however, this might not be the case for populations with a larger number of source populations.

For the third dataset simulating American admixture, Gnomix and FLARE have comparably accurate estimates (Fig. S6—see online supplementary material for a colour version of this figure). FRAME is less accurate than both Gnomix and FLARE for this dataset. This could potentially be due to inadequate IBD-sharing. The differences in the admixture levels of the simulated individuals could also affect this result. Figure S7 (see online supplementary material for a colour version of this figure) shows that 3-way admixture simulated using SLiM has more “noisy”-ly admixed individuals in comparison to the American admixture simulated data. Twenty query individuals were randomly selected for each data set, and their ground truth local ancestry values were visualized for both datasets in Fig. S7 (see online supplementary material for a colour version of this figure).

#### Improvement with site-wise ancestry dosage

In this section, we demonstrate that estimates calculated by aggregating site-wise ancestry dosages yield significantly better accuracy than making explicit local ancestry calls. Figure 2 shows improvements in accuracy for Gnomix and FLARE. Gnomix${\;}^{+}$ and FLARE${\;}^{+}$ represent the aggregated ancestry dosages for these tools. The accuracy improved by ∼73%, reducing the median RMSE value from 0.3344 to 0.0879 for Gnomix. Similarly, accuracy improved by ∼63% for FLARE from a median RMSE of 0.2120 to 0.0783. We observe similar improvements for Gnomix and FLARE for the remaining groups of 3-way datasets (see Supplementary information).

For the 2-way admixed data, the improvement is more pronounced for 80:20 and 70:30 admixed sets. Figure 4 shows one such example where RMSE for Gnomix improved by ∼23%, whereas KL divergence improved by ∼49%. This improvement factor is not as pronounced for FLARE as it improved by ∼1.5% for RMSE and ∼5% for KL divergence value. Across all the 2-way datasets, the RMSE and KL divergence values for Gnomix improved by ∼15–23% and 32–51%, respectively. Similarly, FLARE’s RMSE and KL divergence values improved by ∼0–3% and 0–9%, respectively. While the factor of improvement appears to be influenced by admixture levels, in general, aggregating site-wise ancestry dosages rather than making explicit local ancestry calls improves the accuracy of estimated proportions.

#### Accuracy improvement with panel refinement

We show that we can improve the accuracy of our estimates by refining the reference panel. We do so by increasing the homogeneity of the populations in the reference panel. We show this for the 55:45 2-way admixed set without any genotyping errors. First, we filter the original reference panel to retain more homogeneous individuals. We define such individuals as individuals that are at least 70:30 admixed in either direction of the source populations. There were 66 individuals who met this cut-off for the 55:45 admixed dataset.

**Figure 5:**
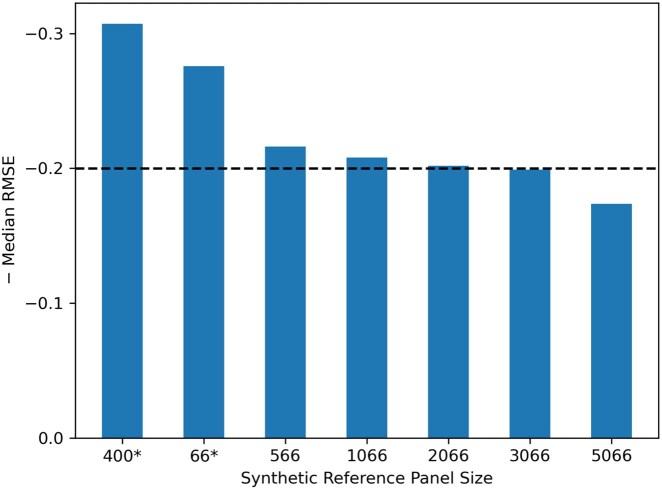
Effect of panel refinement on inferred estimates for 2-way (55:45) admixed data. The dashed line shows the negative median RMSE value for ADMIXTURE’s estimate with the original panel. The $400^{*}$ reference panel is the original reference panel without any synthetic individuals and $66^{*}$ represents the filtered panel retaining homogeneously admixed samples from the original reference panel.

The first two bars with an asterisk (*) in Fig. 5 show the original panel of 400 individuals and the filtered original panel with 66 individuals. The remaining bars show the refined panel consisting of synthetic individuals with the 66 filtered samples. Here, the synthetic individuals were created to have at least 70% contribution from the ancestral population using a window size of 200 sites. The accuracy improves from a median RMSE of 0.3073 to 0.2615 by simply filtering out the non-homogeneous individuals. Furthermore, the accuracy gradually increases with the size of the refined panel. The horizontal dashed line represents ADMIXTURE’s estimate which was the most accurate estimate for this dataset. With a refined panel size of 3066 and above, FRAME is more accurate than ADMIXTURE.

### Evaluation on 1000 Genomes Project data

In this section, we use 1KGP data to show the importance of reference panels in genetic ancestry estimation as well as benchmark runtime and memory usage. We run our tests on a panel of randomly sampled 500 query individuals. We show the changes in FRAME’s estimates when using the 1KGP panel, the synthetic panel, and the HGDP panel as our reference panels. We also evaluate the performance of our method against the state-of-the-art tools.

#### Effect of reference panel

We inferred the genetic ancestry estimates of the randomly chosen 500 query samples using the remaining 2004 1KGP samples as our reference panel. We generally observe that the inferred estimates are in line with our expectations. Here, we use geographical descriptors to describe genetic ancestry following the recommendations in (National Academies of Sciences, Engineering, and Medicine 2023). For instance, we see that individuals belonging to the African continental regions such as Esan in Nigeria (ESN), Gambian in Western Division—Mandinka (GWD), Luhya in Webuye, Kenya (LWK), and Yoruba in Ibadan, Nigeria (YRI) are shown to have 82–87% African ancestry, whereas individuals in regions of high potential admixture such as African Caribbean in Barbados (ACB) and African Ancestry in Southwest USA (ASW) show 60–75% African ancestry and noticeable contributions (>10%) from European as well as American ancestry (Bryc *et al.* 2015).

Similarly, populations in European regions show 43–50% European ancestry, populations in East Asian regions show 61–66% East Asian ancestry, populations in South Asian regions show 44–53% South Asian ancestry. As for samples from the admixed American regions, they are inferred to have majority American and European ancestry. The East Asian contribution found in the Finnish in Finland (FIN) populations is similar to the results obtained in (Browning *et al.* 2023), whereas South Asian ancestry for Iberian populations in Spain (IBS) and Toscani in Italia (TSI) populations could be attributed to the early migration of Roma population to those regions (Martínez-Cruz *et al.* 2016; Aizpurua-Iraola *et al.* 2022). There are, however, some unexpected results. For instance, GWD is shown to have more than 10% American ancestry, whereas populations belonging to the American and East Asian regions are shown to have more than 10% South Asian ancestry (Table S11—see online supplementary material). These results are inconsistent with the results in the current literature. It is been shown that populations in American regions are admixed between European, African, and Native American ancestries (Martínez-Cortés *et al.* 2012; Rishishwar *et al.* 2015; Noel *et al.* 2017) and East Asian populations are not shown to have noticeable South Asian ancestries (Chiang *et al.* 2018; Pan and Xu 2020) with the exception of recent discoveries of South Asian ancestry among some South-East Asian groups (Changmai *et al.* 2022b). Such results could be due to the lack of proper representation and diversity of Asian regions in the 1KGP panel itself (Lu and Xu 2013).

To further evaluate FRAME’s performance, we compared the genome-wide ancestry estimates from FRAME with RFMix and ADMIXTURE. We performed regression of the estimates between FRAME and RFMix and between FRAME and ADMIXTURE for each reference population for all the 500 samples. We observe a strong correlation between FRAME and the two tools for African ($r^{2}\geq 0.99)$ and East Asian ($r^{2}\geq 0.96$) populations (Fig. S8—see online supplementary material for a colour version of this figure). The correlation for European ($r^{2}\geq 0.84)$ and South Asian ($r^{2}\geq 0.89)$ populations are also strong. In comparison, we observe a weaker correlation for American ($r^{2}\geq 0.72)$ populations. These differences could be explained due to presence of individuals with highly admixed ancestry.

**Figure 6:**
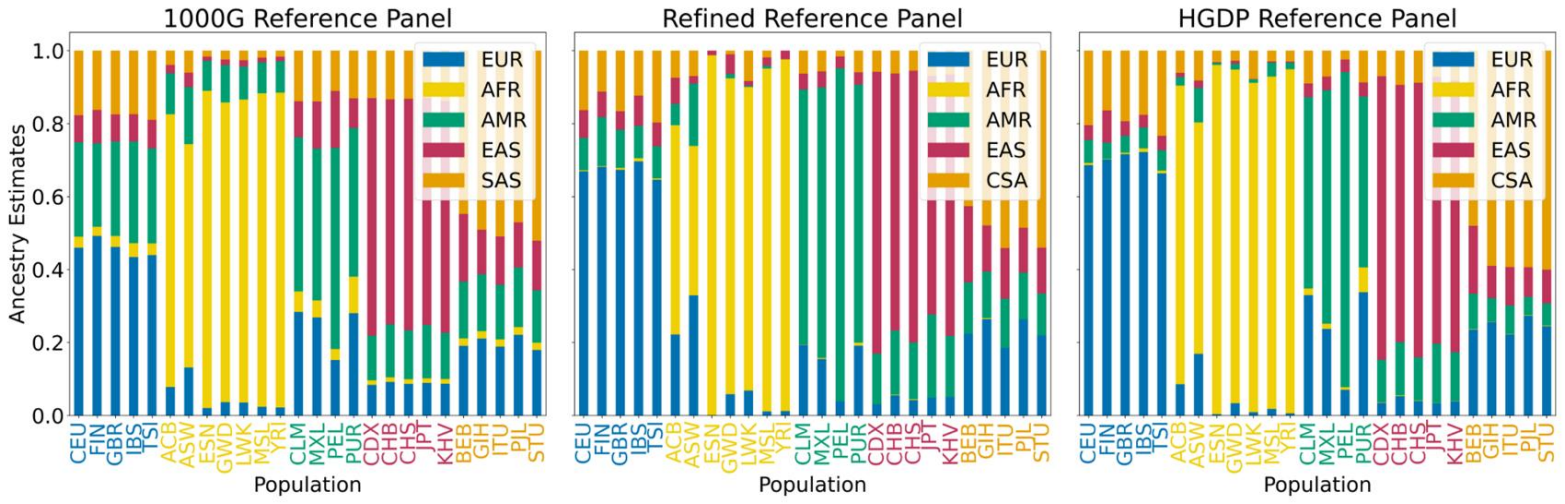
Reference panel affects the ancestry composition estimates. (A) Average ancestry estimates using 1000G as a reference panel. (B) Average ancestry estimates using the refined panel consisting of 2500 samples, 500 samples of each continental population. (C) Average ancestry estimates using the HGDP panel as the reference panel. ACB, African Caribbean in Barbados; ASW, African ancestry in Southwest USA; BEB, Bengali in Bangladesh; CDX, Chinese Dai in Xishuangbanna, China; CEU, Utah residents (CEPH) with Northern and Western European ancestry; CHB, Han Chinese in Beijing, China; CHS, Han Chinese South, China; CLM, Colombian in Medellian, Colombia; ESN, Esan in Nigeria; FIN, Finnish in Finland; GBR, British in England and Scotland; GIH, Gujarati Indians in Houston, Texas, USA; GWD, Gambian in Western Division-Mandinka; IBS, Iberian populations in Spain; ITU, Indian Telugu in the UK; JPT, Japanese in Tokyo, Japan; KHV, Kinh in Ho Chi Minh city, Vietnam; LWK, Luhya in Webuye, Kenya; MSL, Mende in Sierra Leone; MXL, Mexican Ancestry in Los Angeles, California, USA; PEL, Peruvian in Lima, Peru; PUR, Puerto Rican in Puerto Rico; PJL, Punjabi in Lahore, Pakistan; STU, Sri Lankan Tamil in the UK; TSI, Toscani in Italia; YRI, Yoruba in Ibadan, Nigeria.

In the second experiment, we created a refined panel to show the changes in the inferred estimates. We created the refined panel by sampling segments from the 2004 reference samples of the 1KGP data. To do so, we first estimated the local ancestry values of those samples with FLARE using HGDP as the reference panel and posterior probabilities turned on. Next, we created 500 synthetic individuals of each population using the site-wise ancestry dosages obtained from FLARE’s results. The synthetic individuals were created to have at least 70% contribution from the ancestral population using a window size of 200 sites. Majority of the resulting synthetic individuals in each population are assured to be at least 70% of respective ancestry. Figure 6B shows the estimated proportions averaged over each population. More noticeably, the European ancestry contribution increases by ∼15% in populations of European regions. We see similar increases of respective ancestries in populations of East Asia, and America as well. Such increments of respective source populations can be attributed to the homogenous nature of the refined panel. Additionally, we no longer see noticeable American contribution in GWD as well as South Asian contribution in populations of the American and East Asian regions.

In the third experiment, we use the HGDP panel as our reference panel and compare the results. Figure 6C shows the changes in the estimated proportions for each population group. Here, we see comparable results with the estimates obtained using the refined panel. While each of the continental ancestral contributions increases with the HGDP panel, more noticeably, the American ancestral contribution and European ancestral contribution increase 10–30% in American and European populations with respect to the first estimates, respectively. The increase in the American population could be a result of better representation of Native American populations in the HGDP panel, as it includes samples from the Surui, Maya, Karitiana, Pima, and Colombian ancestries. Similarly, the increase in European populations could be explained by the representation of more Western European populations, such as French, Bergamo Italian, Orcadian, etc., in the HGDP panel. We also compared the results of FRAME against FLARE on randomly sampled eight HGDP samples (see Fig. S9). We chose FLARE for comparison because it has been shown to yield better results among recent ancestry estimation tools. For most of the samples, the estimates between FRAME and FLARE are consistent with FRAME showing slightly higher levels of admixture. However, for the Cambodian (EAS) sample (HGDP00714), there are some noticeable differences. Specifically, FRAME shows ∼11% Central South Asian contribution, whereas FLARE infers ∼99% East Asian contribution. While Cambodians are grouped as East Asians in the HGDP dataset, they are South-East Asians and there are studies showing ancestral contribution from South Asian ancestry in such South-East Asian groups (Changmai *et al.* 2022a, 2022b). It is estimated that ∼9% South Asian contribution is evident in present-day Cambodian individuals. In general, we observe that FRAME’s estimates have higher levels of admixture with respect to FLARE’s estimates. Since these are real datasets and we do not have access to the ground truth, we speculate that the true ancestry composition of these individuals lie somewhere in between FRAME’s admixture-leaning estimates and non-admixture-leaning existing tools.

### Runtime and memory usage

We measure the CPU, wall-clock time, and memory usage of all tools for the full pipeline on 500 randomly sampled 1KGP data. FRAME has the lowest peak memory usage, at 0.43 GB, compared to all the global and local ancestry tools. FRAME also has the fastest runtime, both in CPU hours and wall-clock times, compared to the global ancestry estimation tools. In comparison to the local ancestry tools, FRAME still utilizes the least amount of CPU hours but is ∼2.5 times slower than FLARE (Table S12—see online supplementary material).

We also measure the performance of FRAME on chromosome 20 of UK Biobank (UKB) (Bycroft *et al.* 2018) data with 486 936 individuals and 17 197 variants. Four query panels with 100, 500, 1000, and 2000 individuals were created. These individuals were randomly sampled. Five reference panels with 2000, 5000, 10 000, 20 000, and 50 000 individuals were also created by random sampling. The reference panels and query panels consist of mutually exclusive individuals. IBD segments that were at least 500 sites were used for ancestry score calculation. We measured the inference time for each query panel against the different reference panels. Here, the inference time refers to the total time required to call the IBD segments and calculate the ancestry scores. We observe that FRAME has a fast inference time, which grows sublinearly with respect to the reference panel size for various query panel sizes (Fig. S10—see online supplementary material for a colour version of this figure). The average inference time for the query panels with 100, 500, 1000, and 2000 samples across all the reference panel sizes was 0.090, 0.068, 0.062, and 0.063 seconds, respectively. We also measured the peak memory for the overall pipeline and observed that it grows linearly with the reference panel size (Fig. S11—see online supplementary material for a colour version of this figure). This is expected as the panel is precomputed to call the IBDs by Syllable-PBWT. However, the memory footprint remains very low, demonstrating its scalability to biobank-scale panels.

## Discussion

In this work, we have presented an IBD-based method, FRAME, which uses IBD segments to estimate the genetic ancestry composition of individuals. We demonstrated that using only ancestry-informative markers, as covered by the IBD segments, can be a reliable indicator of source population contribution. Our results demonstrate that considering multiple potential ancestries for each segment of an individual can significantly improve the inference of their ancestral composition. We demonstrated improvement in the accuracy of the estimates provided by the benchmarked tools using 2-way and 3-way simulated datasets. While FRAME had the most accurate estimates for 3-way, 80:20 admixed, and 70:30 admixed 2-way datasets, ADMIXTURE performed better for more heterogeneous admixed sets, such as 60:40 and 55:45 2-way admixed sets, and we encourage the users to keep this in mind when using the tool. FRAME also has the best performance for populations that have high levels of IBD sharing, but can perform relatively well in other circumstances, too.

We also bring attention to reference panels and their importance in many applications such as imputation, phasing, and genetic ancestry estimation. However, to the best of our knowledge, refining such panels has not been given much attention. In the context of ancestry estimation, ideally, a reference panel should consist of haplotypes from source populations that are specific to the query individuals. However, in practice, we only have a proxy panel. A low-quality proxy panel could affect the accuracy of the estimated ancestral proportions. Hence, we proposed a method to refine panels by creating synthetic samples representative of the source populations (i.e. samples that better approximate the source haplotypes, assuming the isolated source populations were homogenous). It should be noted that the assumption of homogeneous populations might not always be true, as it depends on the admixture history. We showed that using such refined panels improved the accuracy of FRAME on the 55:45 2-way simulated dataset. We also demonstrated the effect of using the refined panel in real-world data, such as the 1000 Genomes Project data. While it is challenging to attribute the effects of refined panels to real data due to a lack of ground-truth ancestry information, we demonstrated that using refined panels improves the estimates from major ancestral population contributors. While we provided a simple heuristic approach to panel refinement, it could be further improved by incorporating possible recombination events and admixture history to create synthetic samples that better approximate the source populations. Further experiments are warranted to explore such variations and their effects, and it could potentially be a project in its own right.

## Supplementary Material

vbag006_Supplementary_Data

## Data Availability

The source code for FRAME is available at https://github.com/ucfcbb/FRAME.
