# Supplementary material for: FRAME: fast reference-based ancestry makeup estimation tool
Source: Bioinform Adv. 2026 Jan 12;6(1):vbag006. doi: 10.1093/bioadv/vbag006 (PMC12866910; doi:10.1093/bioadv/vbag006)
Supplement: vbag006_Supplementary_Data [file vbag006_supplementary_data.pdf]

Supplementary Material for "FRAME: Fast reference-based ancestry makeup estimation tool"

Pramesh Shakya[1], Ardalan Naseri[2], Degui Zhi[2], and Shaojie Zhang[1]

[1]Department of Computer Science, University of Central Florida, FL, USA
[2]McWilliams School of Biomedical Informatics, University of Texas Health Science Center at Houston, TX, USA

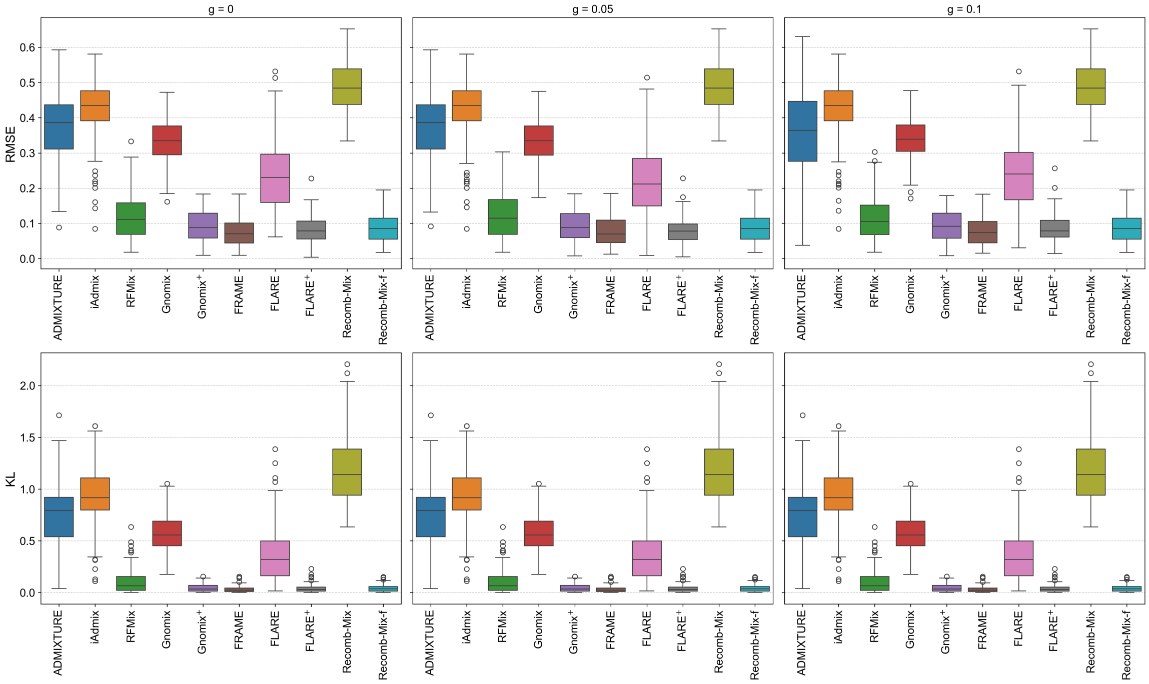

Figure S1: RMSE and KL divergence values for 3-way admixed data at varying genotyping error rates

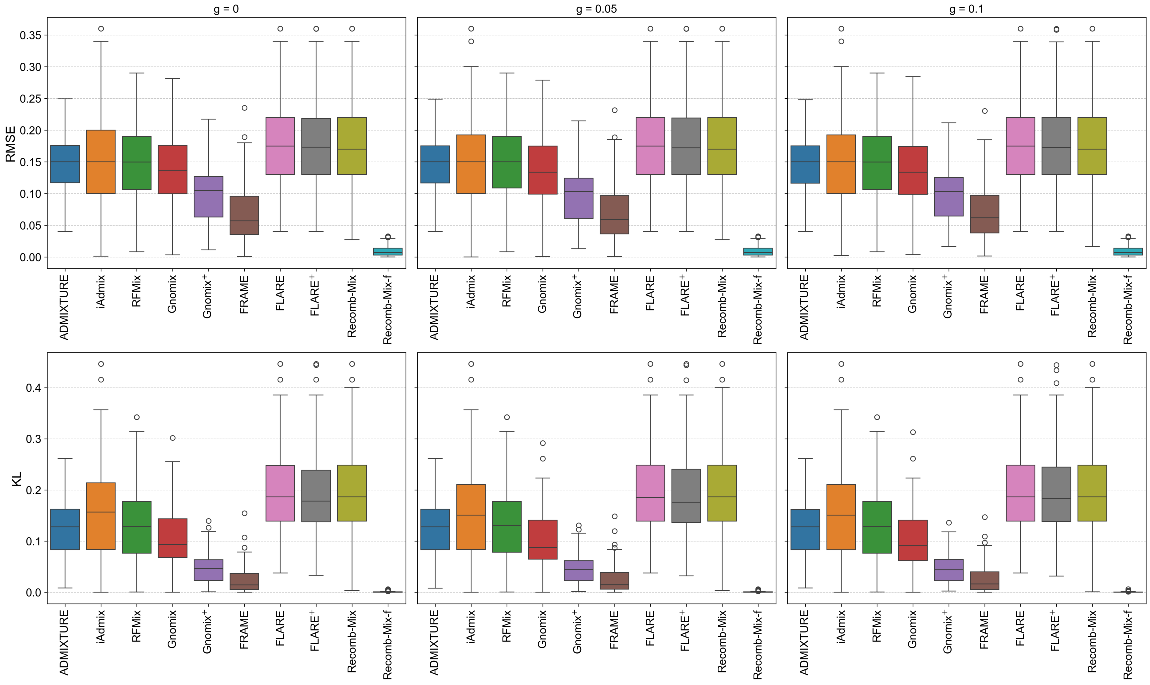

Figure S2: RMSE and KL divergence values for 2-way (80:20) admixed data at varying genotyping error rates

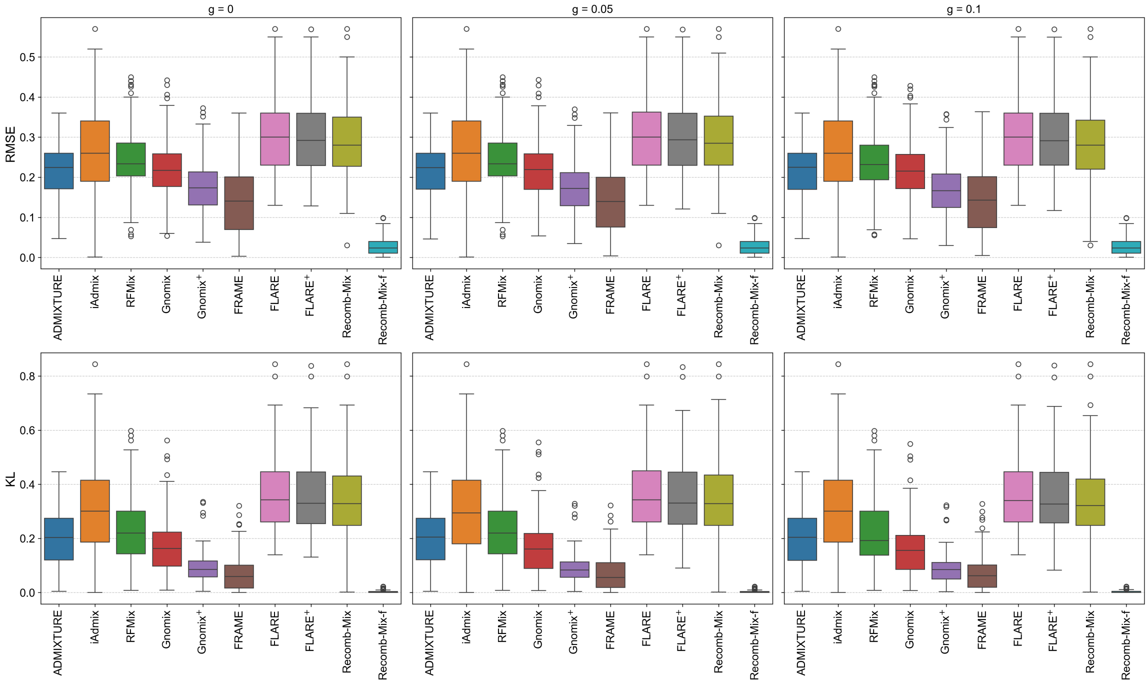

Figure S3: RMSE and KL divergence values for 2-way (70:30) admixed data at varying genotyping error rates

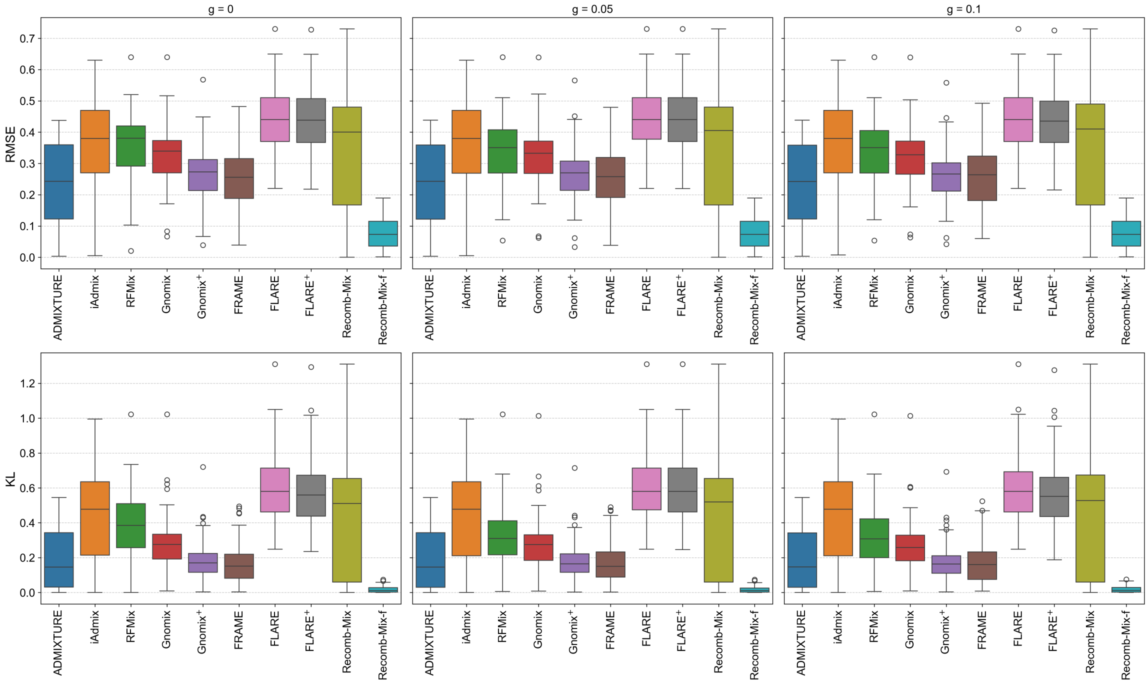

Figure S4: RMSE and KL divergence values for 2-way (60:40) admixed data at varying genotyping error rates

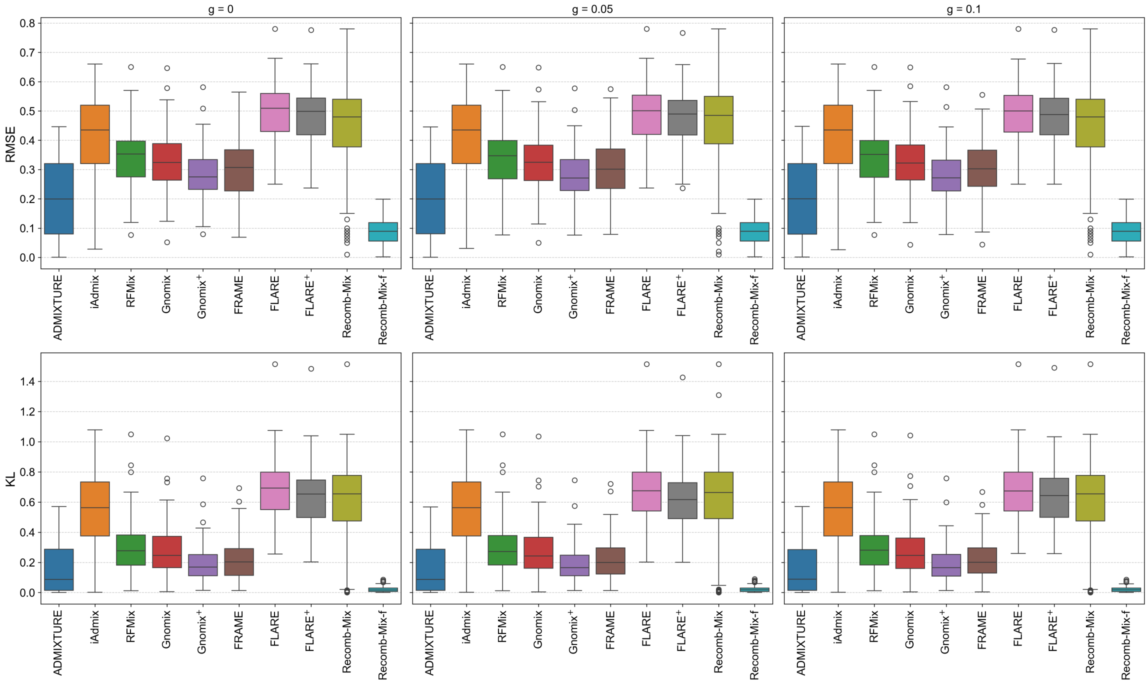

Figure S5: RMSE and KL divergence values for 2-way (55:45) admixed data at varying genotyping error rates

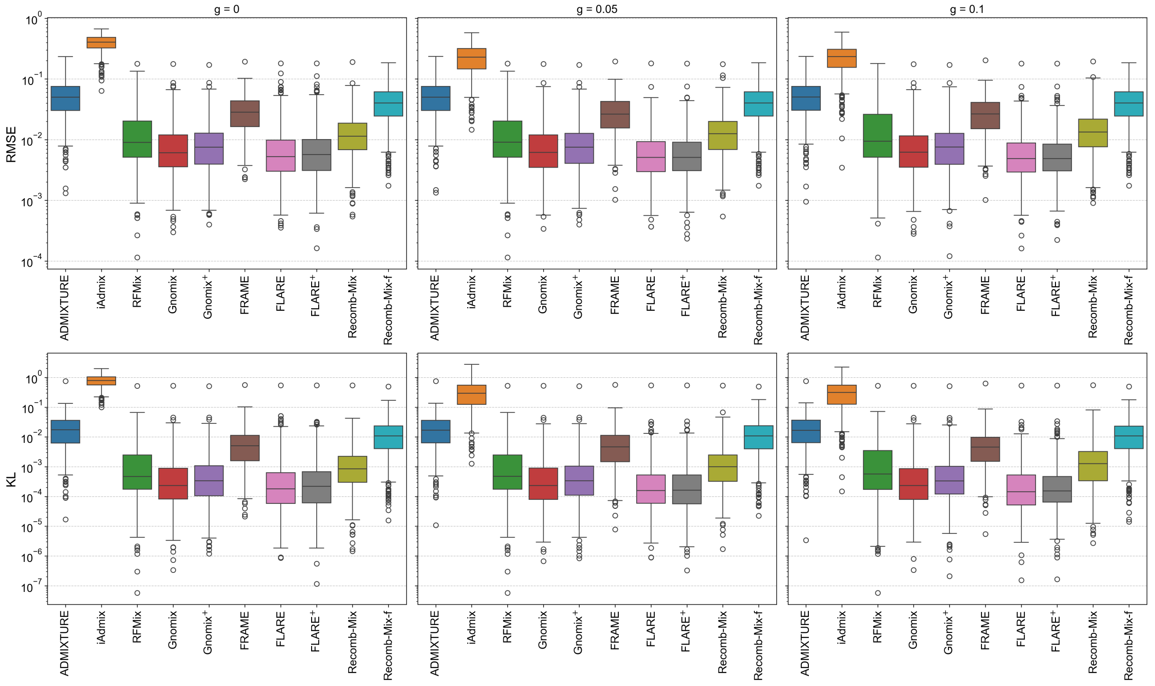

Figure S6: RMSE and KL divergence values for 3-way admixed AmericanAdmixture4B11 data at varying genotyping error rates

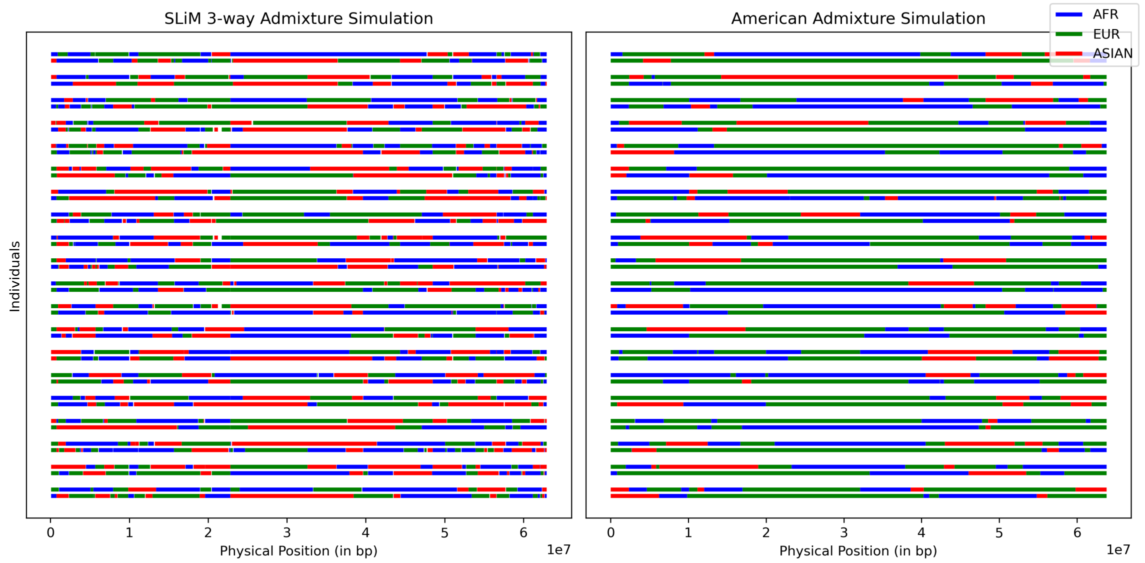

Figure S7: Ground truth admixture levels for 20 randomly sampled query individuals

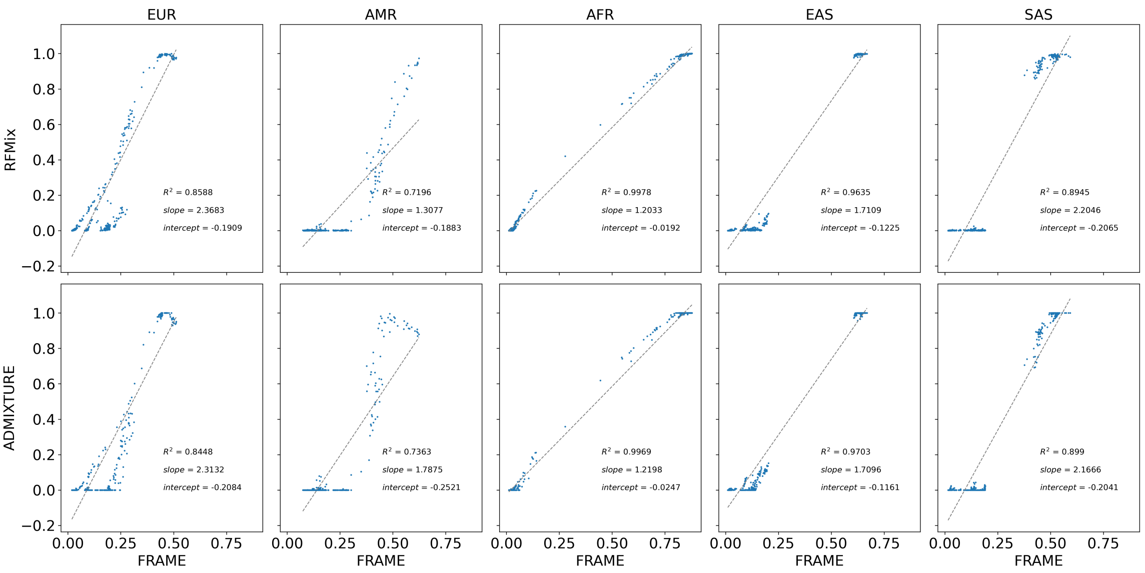

Figure S8: Regression of estimated genome-wide ancestry proportions between FRAME, RFMix and ADMIXTURE on ranomly sampled 500 1KGP query samples

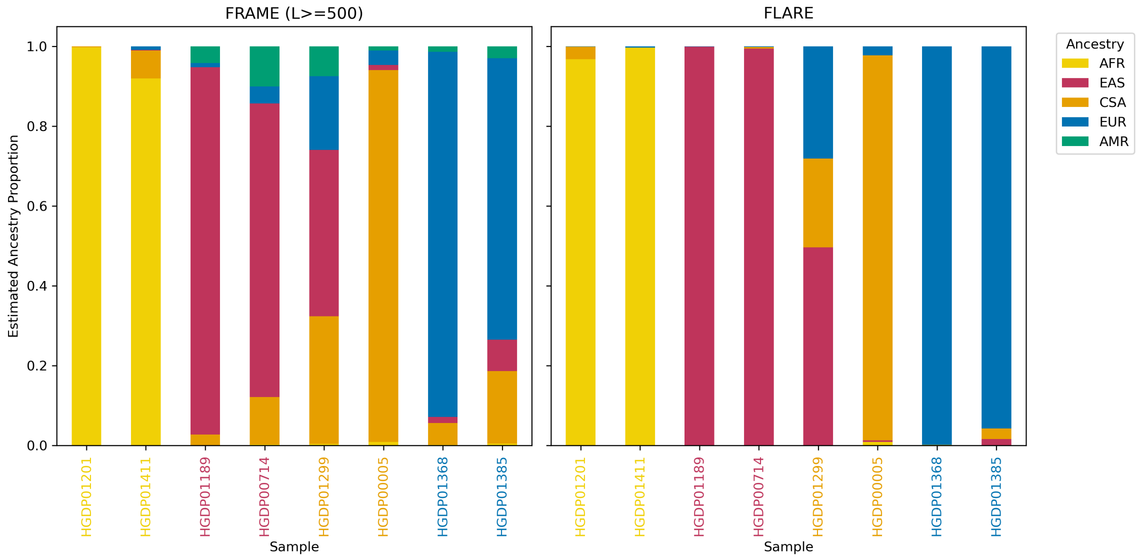

Figure S9: Comparison of FRAME and FLARE on randomly sampled 8 HGDP query samples

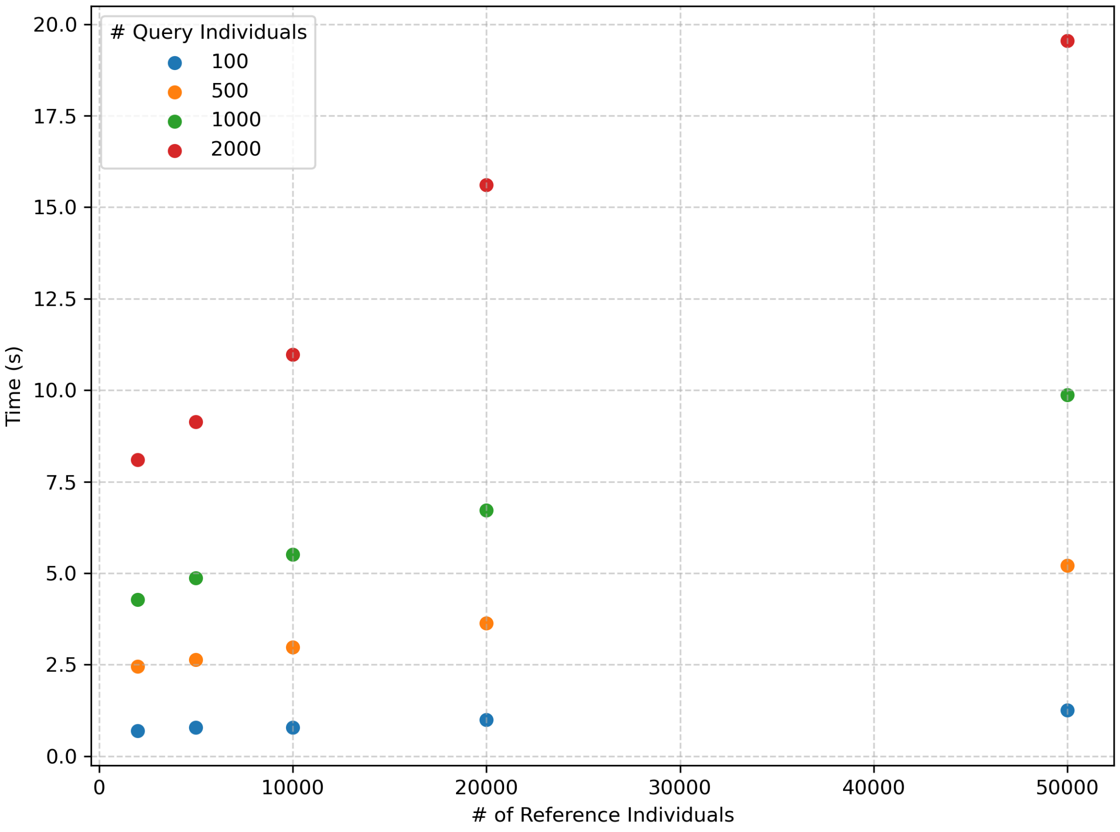

Figure S10: Inference time for various reference panel and query panel sizes on UKB Chr 20 data

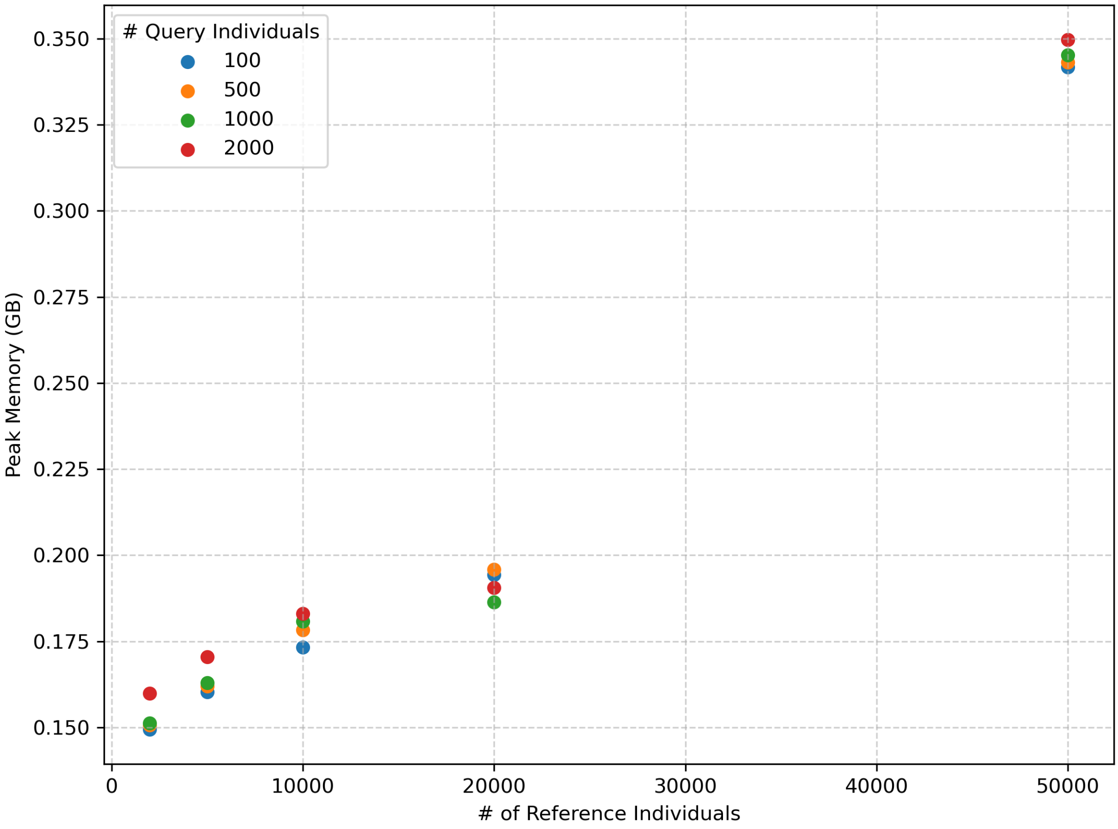

Figure S11: Peak memory usage for various reference panel and query panel sizes on UKB Chr 20 data

Table S1: RMSE for 3-way admixed simulated data using SLiM.

g(%)	Tools	Mean	Median	Min	Max	P-value
0	FRAME	**0.0738**	**0.0704**	0.0093	0.1837	-
	ADMIXTURE	0.3702	0.3865	0.0887	0.5926	3.22E-70*
	iAdmix	0.4195	0.4346	0.0849	0.5806	1.97E-85*
	RFMix	0.1234	0.1114	0.0179	0.3330	3.00E-09*
	Gnomix	0.3362	0.3346	0.1617	0.4723	1.07E-90*
	Gnomix+	0.0909	0.0882	0.0092	0.1833	0.0031*
	FLARE	0.2372	0.2301	0.0615	0.5316	1.40E-33*
	FLARE+	0.0823	0.0787	0.0037	0.2278	0.1218
	Recomb-Mix	0.4884	0.4843	0.3344	0.6521	3.89E-18*
	Recomb-Mix (f=0.1)	0.0870	0.0851	0.0176	0.1952	0.0177*
0.05	FRAME	**0.0763**	**0.0698**	0.0126	0.1849	-
	ADMIXTURE	0.3706	0.3865	0.0916	0.5926	6.76E-69*
	iAdmix	0.4183	0.4346	0.0849	0.5806	1.98E-82*
	RFMix	0.1201	0.1150	0.0179	0.3029	5.91E-08*
	Gnomix	0.3370	0.3344	0.1731	0.4748	1.52E-88*
	Gnomix+	0.0909	0.0879	0.0079	0.1840	0.0153*
	FLARE	0.2237	0.2120	0.0089	0.5141	7.68E-30*
	FLARE+	0.0813	0.0783	0.0051	0.2280	0.3859
	Recomb-Mix	0.4884	0.4843	0.3344	0.6521	3.89E-18*
	Recomb-Mix (f=0.1)	0.0888	0.0840	0.0253	0.1969	0.0280*
0.1	FRAME	**0.0764**	**0.0737**	0.0152	0.1829	-
	ADMIXTURE	0.3554	0.3639	0.0378	0.6304	5.15E-54*
	iAdmix	0.4178	0.4346	0.0849	0.5806	2.60E-82*
	RFMix	0.1178	0.1053	0.0179	0.3029	1.90E-07*
	Gnomix	0.3414	0.3392	0.1711	0.4772	2.58E-90*
	Gnomix+	0.0919	0.0917	0.0080	0.1793	0.0087*
	FLARE	0.2376	0.2402	0.0305	0.5316	1.09E-36*
	FLARE+	0.0857	0.0789	0.0141	0.2566	0.1132
	Recomb-Mix	0.4884	0.4843	0.3344	0.6521	3.89E-18*
	Recomb-Mix (f=0.1)	0.0908	0.0892	0.0221	0.2451	0.0418*

* P-value < 0.05. Bold values show the lowest mean and median values

Table S2: KL divergence for 3-way admixed simulated data using SLiM.

g(%)	Tools	Mean	Median	Min	Max	P-value
0	FRAME	**0.0329**	**0.0229**	0.0004	0.1552	-
	ADMIXTURE	0.7546	0.7922	0.0377	1.7145	1.29E-59*
	iAdmix	0.9244	0.9163	0.1096	1.6094	3.50E-75*
	RFMix	0.1125	0.0661	0.0015	0.6349	4.66E-09*
	Gnomix	0.5800	0.5566	0.1762	1.0525	1.48E-77*
	Gnomix+	0.0455	0.0339	0.0005	0.1550	0.0135*
	FLARE	0.3676	0.3192	0.0164	1.3863	7.26E-25*
	FLARE+	0.0403	0.0281	0.0005	0.2300	0.1552
	Recomb-Mix	1.1990	1.1394	6.35E-01	2.2073	3.89E-18*
	Recomb-Mix (f=0.1)	0.0427	0.0354	1.32E-03	0.1510	0.0300*
0.05	FRAME	**0.0363**	**0.0255**	0.0008	0.2237	-
	ADMIXTURE	0.7552	0.7921	0.0403	1.7145	2.99E-59*
	iAdmix	0.9208	0.9163	0.1212	1.6094	2.98E-73*
	RFMix	0.1044	0.0660	0.0015	0.4913	2.12E-08*
	Gnomix	0.5867	0.5582	0.1884	1.0633	1.97E-77*
	Gnomix+	0.0457	0.0341	0.0004	0.1562	0.0858
	FLARE	0.3325	0.2809	0.0003	1.2040	2.37E-24*
	FLARE+	0.0395	0.0291	0.0004	0.2313	0.5662
	Recomb-Mix	1.1990	1.1394	6.35E-01	2.2073	3.89E-18*
	Recomb-Mix (f=0.1)	0.0435	0.0353	0.0032	0.1538	0.0837
0.1	FRAME	**0.0352**	**0.0248**	0.0010	0.1659	-
	ADMIXTURE	0.7282	0.7124	0.0071	2.2070	1.70E-43*
	iAdmix	0.9198	0.9163	0.1039	1.6094	4.31E-73*
	RFMix	0.1006	0.0616	0.0015	0.4913	3.83E-08*
	Gnomix	0.5891	0.5645	0.1862	1.0807	1.33E-76*
	Gnomix+	0.0463	0.0370	0.0004	0.1487	0.0333*
	FLARE	0.3620	0.3466	0.0041	1.3863	1.24E-29*
	FLARE+	0.0443	0.0285	0.0003	0.2864	0.1179
	Recomb-Mix	1.1990	1.1394	6.35E-01	2.2073	3.89E-18*
	Recomb-Mix (f=0.1)	0.0473	0.0358	0.0027	0.2685	0.0604

* P-value < 0.05. Bold values show the lowest mean and median values

Table S3: RMSE for 2-way 80:20 admixed simulated data.

g(%)	Tools	Mean	Median	Min	Max	P-value
0	FRAME	0.0711	0.0571	0.0018	0.2354	-
	ADMIXTURE	0.1436	0.1500	0.0400	0.2494	8.35E-22*
	iAdmix	0.1516	0.1500	0.0014	0.3600	1.40E-15*
	RFMix	0.1480	0.1499	0.008	0.2900	7.09E-20*
	Gnomix	0.1405	0.1369	0.0035	0.2817	1.01E-17*
	Gnomix+	0.1026	0.1049	0.0112	0.2174	8.31E-06*
	FLARE	0.1771	0.1750	0.0400	0.3600	8.48E-27*
	FLARE+	0.1763	0.1732	0.0400	0.3600	1.72E-26*
	Recomb-Mix	0.1772	0.1700	0.0273	0.3600	4.13e-18*
	Recomb-Mix (f=0.1)	**0.0103**	**0.0074**	2.00E-06	0.0329	9.60E-18*
0.05	FRAME	0.0725	0.0592	0.0008	0.2316	-
	ADMIXTURE	0.1435	0.1500	0.0400	0.2490	9.72E-21*
	iAdmix	0.1509	0.1500	0.0000	0.3600	7.91E-15*
	RFMix	0.1490	0.1500	0.0084	0.2900	1.28E-19*
	Gnomix	0.1384	0.1338	0.0011	0.2789	2.45E-16*
	Gnomix+	0.1006	0.1031	0.0131	0.2148	7.47E-05*
	FLARE	0.1770	0.1750	0.0400	0.3600	5.71E-26*
	FLARE+	0.1760	0.1724	0.0400	0.3600	1.24E-25*
	Recomb-Mix	0.1772	0.1700	0.0273	0.3600	4.26E-18*
	Recomb-Mix (f=0.1)	**0.0108**	**0.0081**	0.0000	0.0317	1.64E-17*
0.1	FRAME	0.0741	0.0619	0.0017	0.2305	-
	ADMIXTURE	0.1431	0.1500	0.0400	0.2481	2.50E-20*
	iAdmix	0.1506	0.1500	0.0025	0.3600	2.05E-14*
	RFMix	0.1479	0.1499	0.0084	0.2900	1.04E-18*
	Gnomix	0.1386	0.1338	0.0039	0.2844	4.21E-16*
	Gnomix+	0.1006	0.1031	0.0167	0.2117	0.0001*
	FLARE	0.1775	0.1750	0.0400	0.3600	8.96E-26*
	FLARE+	0.1767	0.1729	0.0400	0.3598	1.44E-25*
	Recomb-Mix	0.1758	0.1700	0.0169	0.3600	9.31E-18*
	Recomb-Mix (f=0.1)	**0.0099**	**0.0070**	2.00E-06	0.0344	5.76E-18*

* P-value < 0.05. Bold values show the lowest mean and median values

Table S4: KL divergence for 2-way 80:20 admixed simulated data

g(%)	Tools	Mean	Median	Min	Max	P-value
0	FRAME	0.0253	0.0144	0.0000	0.1544	-
	ADMIXTURE	0.1241	0.1279	0.0083	0.2613	2.71E-37*
	iAdmix	0.1577	0.1567	0.0000	0.4463	1.09E-26*
	RFMix	0.1352	0.1283	0.0006	0.3425	4.23E-30*
	Gnomix	0.1081	0.0933	0.0001	0.3018	3.26E-28*
	Gnomix+	0.0490	0.0468	0.0009	0.1395	8.72E-08*
	FLARE	0.1949	0.1863	0.0378	0.4463	1.00E-45*
	FLARE+	0.1909	0.1782	0.0331	0.4463	1.52E-44*
	Recomb-Mix	0.1972	0.1863	3.52E-03	0.4463	4.01E-18*
	Recomb-Mix (f=0.1)	**0.0007**	**0.0002**	1.1618e-11	0.0062	9.89E-18*
0.05	FRAME	0.0265	0.0148	0.0000	0.1487	-
	ADMIXTURE	0.1238	0.1277	0.0082	0.2613	2.96E-36*
	iAdmix	0.1562	0.1508	0.0000	0.4463	9.28E-26*
	RFMix	0.1368	0.1310	0.0006	0.3425	1.75E-30*
	Gnomix	0.1043	0.0878	0.0000	0.2915	1.79E-26*
	Gnomix+	0.0469	0.0450	0.0012	0.1310	3.15E-06*
	FLARE	0.1947	0.1855	0.0378	0.4463	4.40E-45*
	FLARE+	0.1899	0.1760	0.0320	0.4463	7.80E-44*
	Recomb-Mix	0.1972	0.1863	3.52E-03	0.4463	4.01E-18*
	Recomb-Mix (f=0.1)	**0.0007**	**0.0002**	1.1618e-11	0.0062	1.29E-17*
0.1	FRAME	0.0273	0.0163	0.0000	0.1469	-
	ADMIXTURE	0.1229	0.1277	0.0085	0.2613	1.10E-35*
	iAdmix	0.1559	0.1508	0.0000	0.4463	2.28E-25*
	RFMix	0.1352	0.1283	0.0006	0.3425	3.49E-29*
	Gnomix	0.1047	0.0910	0.0001	0.3131	7.41E-26*
	Gnomix+	0.0467	0.0443	0.0023	0.1362	7.39E-06*
	FLARE	0.1962	0.1863	0.0377	0.4463	3.67E-45*
	FLARE+	0.1928	0.1837	0.0319	0.4443	1.96E-44*
	Recomb-Mix	0.1960	0.1863	9.88E-04	0.4463	4.39E-18*
	Recomb-Mix (f=0.1)	**0.0006**	**0.0002**	1.1618e-11	0.0062	6.90E-18*

* P-value < 0.05. Bold values show the lowest mean and median values

Table S5: RMSE for 2-way 70:30 admixed simulated data.

g(%)	Tools	Mean	Median	Min	Max	P-value
0	FRAME	0.1468	0.1407	0.0028	0.3603	-
	ADMIXTURE	0.2174	0.2244	0.0472	0.3600	1.92E-09*
	iAdmix	0.2662	0.2600	0.0010	0.5700	6.24E-14*
	RFMix	0.2478	0.2338	0.0526	0.4500	2.82E-14*
	Gnomix	0.2227	0.2174	0.0538	0.4420	4.97E-10*
	Gnomix+	0.1751	0.1738	0.0380	0.3725	0.0108*
	FLARE	0.3032	0.300	0.1300	0.5700	2.22E-25*
	FLARE+	0.3018	0.2918	0.1286	0.5693	5.03E-25*
	Recomb-Mix	0.2897	0.2800	0.0300	0.5700	5.17E-17*
	Recomb-Mix (f=0.1)	**0.0282**	**0.0237**	0.0003	0.0989	6.12E-18*
0.05	FRAME	0.1478	0.1395	0.0040	0.3608	-
	ADMIXTURE	0.2173	0.2238	0.0462	0.0462	3.04E-09*
	iAdmix	0.2652	0.2600	0.0011	0.5700	1.36E-13*
	RFMix	0.2478	0.2338	0.0526	0.4500	4.37E-14*
	Gnomix	0.2209	0.2194	0.0538	0.4429	2.01E-09*
	Gnomix+	0.1728	0.1722	0.0346	0.3695	0.0236*
	FLARE	0.3037	0.300	0.1300	0.5700	2.53E-25*
	FLARE+	0.3017	0.2935	0.1208	0.5687	8.42E-25*
	Recomb-Mix	0.2931	0.2847	0.0300	0.5700	5.17E-17*
	Recomb-Mix (f=0.1)	**0.0284**	**0.0228**	0.0002	0.0985	6.12E-18*
0.1	FRAME	0.1486	0.1429	0.0048	0.3634	-
	ADMIXTURE	0.2171	0.2251	0.0472	0.3600	6.96E-09*
	iAdmix	0.2661	0.2600	0.0009	0.5700	1.80E-13*
	RFMix	0.2448	0.2316	0.0551	0.4500	4.62E-13*
	Gnomix	0.2181	0.2153	0.0464	0.4281	1.26E-08*
	Gnomix+	0.1699	0.1662	0.0300	0.3577	0.0549
	FLARE	0.3026	0.300	0.1300	0.5700	1.64E-24*
	FLARE+	0.3012	0.2913	0.1169	0.569	4.06E-24*
	Recomb-Mix	0.2815	0.2800	0.0300	0.5700	8.47E-15*
	Recomb-Mix (f=0.1)	**0.0294**	**0.0238**	0.0002	0.0989	7.55E-18*

* P-value < 0.05. Bold values show the lowest mean and median values

Table S6: KL divergence for 2-way 70:30 admixed simulated data.

g(%)	Tools	Mean	Median	Min	Max	P-value
0	FRAME	0.0736	0.0589	0.0000	0.3210	-
	ADMIXTURE	0.1976	0.2035	0.0045	0.4462	1.22E-17*
	iAdmix	0.3074	0.3011	0.0000	0.8440	7.22E-25*
	RFMix	0.2326	0.2197	0.0083	0.5978	3.34E-21*
	Gnomix	0.1777	0.1629	0.0089	0.5621	5.16E-14*
	Gnomix+	0.0967	0.0859	0.0049	0.3359	0.0152*
	FLARE	0.3664	0.3425	0.1393	0.8440	7.42E-44*
	FLARE+	0.3589	0.3301	0.1311	0.8378	1.67E-42*
	Recomb-Mix	0.3446	0.3285	1.80E-03	0.8440	6.31E-18*
	Recomb-Mix (f=0.1)	**0.0032**	**0.0013**	3.6035e-07	0.0222	7.33E-18*
0.05	FRAME	0.0746	0.0556	0.0000	0.3221	-
	ADMIXTURE	0.1974	0.2051	0.0043	0.4462	1.99E-17*
	iAdmix	0.3050	0.2944	0.0000	0.8440	2.58E-24*
	RFMix	0.2326	0.2197	0.0083	0.5978	4.92E-21*
	Gnomix	0.1760	0.1608	0.0076	0.5550	3.45E-13*
	Gnomix+	0.0941	0.0831	0.0041	0.3283	0.0376*
	FLARE	0.3682	0.3425	0.1393	0.8440	3.20E-44*
	FLARE+	0.3586	0.3305	0.0908	0.8335	2.19E-42*
	Recomb-Mix	0.3497	0.3285	1.80E-03	0.8440	5.43E-18*
	Recomb-Mix (f=0.1)	**0.0033**	**0.0012**	1.0322e-07	0.0222	5.76E-18*
0.1	FRAME	0.0761	0.0623	0.0001	0.3279	-
	ADMIXTURE	0.1969	0.2045	0.0045	0.4462	1.16E-16*
	iAdmix	0.3072	0.3011	0.0000	0.8440	3.23E-24*
	RFMix	0.2265	0.1922	0.0083	0.5978	4.21E-19*
	Gnomix	0.1707	0.1561	0.0075	0.5492	5.58E-12*
	Gnomix+	0.0910	0.0849	0.0030	0.3229	0.1186
	FLARE	0.3649	0.3402	0.1393	0.8440	2.45E-42*
	FLARE+	0.3573	0.3273	0.0830	0.8391	6.69E-41*
	Recomb-Mix	0.3297	0.3216	1.80E-03	0.8440	6.34E-17*
	Recomb-Mix (f=0.1)	**0.0035**	**0.0013**	1.0322e-07	0.0222	8.02E-18*

* P-value $<$ 0.05. Bold values show the lowest mean and median values

Table S7: RMSE for 2-way 60:40 admixed simulated data.

g(%)	Tools	Mean	Median	Min	Max	P-value
0	FRAME	0.2570	0.2560	0.0393	0.4820	-
	ADMIXTURE	0.2286	0.2431	0.0036	0.4379	0.4290
	iAdmix	0.3586	0.3800	0.0049	0.6300	6.32E-10*
	RFMix	0.3624	0.3809	0.0207	0.6400	7.42E-17*
	Gnomix	0.3274	0.3392	0.0667	0.6400	7.67E-11*
	Gnomix+	0.2697	0.2730	0.0390	0.5678	0.0212*
	FLARE	0.4434	0.4400	0.2200	0.7300	4.16E-34*
	FLARE+	0.4387	0.4381	0.2177	0.7280	1.91E-33*
	Recomb-Mix	0.3447	0.4000	0.0000	0.7300	0.0005*
	Recomb-Mix (f=0.1)	**0.0766**	**0.0735**	0.0013	0.1896	4.26E-18*
0.05	FRAME	1.2606	0.2576	0.0381	0.4798	-
	ADMIXTURE	0.2284	0.2428	0.0033	0.4382	0.0531
	iAdmix	0.3585	0.3800	0.0051	0.6300	2.92E-07*
	RFMix	0.3394	0.3507	0.0536	0.6400	2.92E-08*
	Gnomix	0.3254	0.3327	0.0621	0.6391	1.70E-06*
	Gnomix+	0.2677	0.2703	0.0331	0.5658	0.5745
	FLARE	0.4445	0.4400	0.2200	0.7300	1.84E-28*
	FLARE+	0.4440	0.4400	0.2197	0.7300	2.37E-28*
	Recomb-Mix	0.3457	0.4050	0.0000	0.7300	0.0005*
	Recomb-Mix (f=0.1)	**0.0763**	**0.0735**	0.0013	0.1896	4.01E-18*
0.1	FRAME	0.2600	0.2637	0.0596	0.4922	-
	ADMIXTURE	0.2285	0.2423	0.0036	0.4386	0.0570
	iAdmix	0.3586	0.3800	0.0074	0.6300	2.52E-07*
	RFMix	0.3395	0.3507	0.0536	0.6400	2.70E-08*
	Gnomix	0.3218	0.3278	0.0637	0.6391	4.28E-06*
	Gnomix+	0.2641	0.2663	0.0422	0.5585	0.7487
	FLARE	0.4419	0.4400	0.2200	0.7300	2.57E-28*
	FLARE+	0.4351	0.4355	0.2152	0.7255	5.94E-27*
	Recomb-Mix	0.3549	0.4100	0.0000	0.7300	0.0001*
	Recomb-Mix (f=0.1)	**0.0772**	**0.0746**	0.0008	0.1898	4.01E-18*

* P-value < 0.05. Bold values show the lowest mean and median values

Table S8: KL divergence for 2-way 60:40 admixed simulated data.

g(%)	Tools	Mean	Median	Min	Max	P-value
0	FRAME	0.1697	0.1514	0.0034	0.4944	-
	ADMIXTURE	0.1968	0.1463	0.0000	0.5446	0.0189*
	iAdmix	0.4390	0.4780	0.0000	0.9943	2.20E-19*
	RFMix	0.3785	0.3856	0.0009	1.0217	1.48E-23*
	Gnomix	0.2848	0.2755	0.0098	1.0217	2.23E-13*
	Gnomix+	0.1817	0.1697	0.0033	0.7207	0.0220*
	FLARE	0.5985	0.5798	0.2485	1.3093	3.07E-51*
	FLARE+	0.5734	0.5589	0.2351	1.2929	7.61E-50*
	Recomb-Mix	0.4443	0.5108	0.00	1.3093	4.05E-12*
	Recomb-Mix (f=0.1)	**0.0175**	**0.0114**	4.04E-06	0.0748	4.39E-18*
0.05	FRAME	0.1741	0.1501	0.0032	0.4895	-
	ADMIXTURE	0.1964	0.1461	0.0000	0.5446	0.2908
	iAdmix	0.4389	0.4780	0.0001	0.9943	2.06E-16*
	RFMix	0.3195	0.3105	0.0060	1.0217	5.53E-12*
	Gnomix	0.2817	0.2751	0.0085	1.0137	1.32E-08*
	Gnomix+	0.1790	0.1650	0.0024	0.7147	0.7514
	FLARE	0.6038	0.5798	0.2485	1.3093	2.86E-47*
	FLARE+	0.6007	0.5798	0.2458	1.3093	6.93E-47*
	Recomb-Mix	0.4454	0.5192	0.00	1.3093	7.08E-12*
	Recomb-Mix (f=0.1)	**0.0172**	**0.0118**	4.04E-06	0.0748	4.13E-18*
0.1	FRAME	0.1736	0.1607	0.0086	0.5243	-
	ADMIXTURE	0.1964	0.1471	0.0000	0.5446	0.2798
	iAdmix	0.4389	0.4780	0.0001	0.9943	2.02E-16*
	RFMix	0.3205	0.3077	0.0060	1.0217	6.80E-12*
	Gnomix	0.2737	0.2582	0.0088	1.0137	7.82E-08*
	Gnomix+	0.1736	0.1632	0.0039	0.6922	0.9984
	FLARE	0.5919	0.5798	0.2485	1.3093	1.90E-46*
	FLARE+	0.5593	0.5515	0.1878	1.2761	5.02E-43*
	Recomb-Mix	0.4605	0.5276	0.00	1.3093	3.86E-12*
	Recomb-Mix (f=0.1)	**0.0179**	**0.0124**	1.68E-06	0.0749	4.13E-18*

* P-value < 0.05. Bold values show the lowest mean and median values

Table S9: RMSE for 2-way 55:45 admixed simulated data.

g(%)	Tools	Mean	Median	Min	Max	P-value
0	FRAME	0.2981	0.3073	0.0690	0.5642	-
	ADMIXTURE	0.2021	0.1998	0.0005	0.4465	0.0005*
	iAdmix	0.4019	0.4350	0.0279	0.6600	8.30E-12*
	RFMix	0.3415	0.3533	0.0767	0.6500	1.19E-08*
	Gnomix	0.3273	0.3242	0.0517	0.6463	4.88E-06*
	Gnomix+	0.2793	0.2752	0.0791	0.5813	0.1826
	FLARE	0.4929	0.5093	0.2500	0.7800	1.12E-40*
	FLARE+	0.4839	0.4989	0.2371	0.7761	3.97E-39*
	Recomb-Mix	0.4363	0.4800	0.0100	0.7800	5.77E-08*
	Recomb-Mix (f=0.1)	**0.0890**	**0.0890**	0.0020	0.1990	4.26E-18*
0.05	FRAME	0.2980	0.3009	0.0784	0.5748	-
	ADMIXTURE	0.2020	0.1998	0.0003	0.4457	6.91E-08*
	iAdmix	0.4020	0.4350	0.0307	0.6600	3.12E-07*
	RFMix	0.3409	0.3471	0.0767	0.6500	0.0023*
	Gnomix	0.3255	0.3251	0.0498	0.6481	0.0574
	Gnomix+	0.2771	0.2713	0.0761	0.5774	0.1309
	FLARE	0.4903	0.5005	0.2370	0.7800	2.37E-30*
	FLARE+	0.4808	0.4894	0.2360	0.7666	6.10E-29*
	Recomb-Mix	0.4439	0.4850	0.0100	0.7800	1.14E-08*
	Recomb-Mix (f=0.1)	**0.0894**	**0.0912**	0.0020	0.2033	4.01E-18*
0.1	FRAME	0.2999	0.3026	0.0438	0.5549	-
	ADMIXTURE	0.2021	0.2001	0.0015	0.4476	4.41E-08*
	iAdmix	0.4022	0.4350	0.0265	0.6600	4.69E-07*
	RFMix	0.3409	0.3517	0.0767	0.6500	0.0037*
	Gnomix	0.3273	0.3222	0.0433	0.6491	0.0584
	Gnomix+	0.2774	0.2716	0.0779	0.5814	0.1042
	FLARE	0.4903	0.5000	0.2500	0.7800	7.21E-30*
	FLARE+	0.4828	0.4875	0.2500	0.7771	1.75E-28*
	Recomb-Mix	0.4363	0.4800	0.0100	0.7800	6.11E-08*
	Recomb-Mix (f=0.1)	**0.0902**	**0.0910**	0.0039	0.1990	4.13E-18*

* P-value < 0.05. Bold values show the lowest mean and median values

Table S10: KL divergence for 2-way 55:45 admixed simulated data.

g(%)	Tools	Mean	Median	Min	Max	P-value
0	FRAME	0.2171	0.2033	0.0136	0.6920	-
	ADMIXTURE	0.1572	0.0878	0.0000	0.5705	0.5643
	iAdmix	0.5284	0.5621	0.0019	1.0788	2.03E-23*
	RFMix	0.2981	0.2772	0.0118	1.0498	1.05E-09*
	Gnomix	0.2760	0.2460	0.0054	1.0231	3.89E-07*
	Gnomix+	0.1917	0.1683	0.0143	0.7570	0.1758
	FLARE	0.6809	0.6931	0.2555	1.5141	1.42E-56*
	FLARE+	0.6371	0.6527	0.2027	1.4837	3.70E-53*
	Recomb-Mix	0.5984	0.6539	2.00E-04	1.5141	9.19E-15*
	Recomb-Mix (f=0.1)	**0.0207**	**0.0173**	8.14E-06	0.0863	4.26E-18*
0.05	FRAME	0.2167	0.1996	0.0128	0.7206	-
	ADMIXTURE	0.1571	0.0877	0.0000	0.5671	0.0070*
	iAdmix	0.5282	0.5621	0.0019	1.0788	4.13E-18*
	RFMix	0.2971	0.2719	0.0118	1.0498	0.0002*
	Gnomix	0.2727	0.2426	0.0050	1.0352	0.0104*
	Gnomix+	0.1890	0.1647	0.0132	0.7447	0.1307
	FLARE	0.6714	0.6754	0.2024	1.5141	1.17E-45*
	FLARE+	0.6269	0.6164	0.2001	1.4261	8.36E-43*
	Recomb-Mix	0.6158	0.6636	2.00E-04	1.5141	5.33E-15*
	Recomb-Mix (f=0.1)	**0.0211**	**0.0182**	8.14E-06	0.0900	4.01E-18*
0.1	FRAME	0.2196	0.2007	0.0040	0.6675	-
	ADMIXTURE	0.1574	0.0881	0.0000	0.5704	0.0049*
	iAdmix	0.5286	0.5621	0.0014	1.0788	6.00E-18*
	RFMix	0.2973	0.2811	0.0118	1.0498	0.0004*
	Gnomix	0.2754	0.2465	0.0038	1.0419	0.0105*
	Gnomix+	0.1889	0.1654	0.0139	0.7574	0.0938
	FLARE	0.6728	0.6733	0.2595	1.5141	1.84E-45*
	FLARE+	0.6347	0.6426	0.2583	1.4908	4.44E-42*
	Recomb-Mix	0.5984	0.6539	2.00e-04	1.5141	1.37E-14*
	Recomb-Mix (f=0.1)	**0.0211**	**0.0188**	3.03E-05	0.0863	4.13E-18*

* P-value $<$ 0.05; Bold values show the lowest mean and median values

Table S11: Estimated ancestry proportions of 500 query samples of 1000 Genomes Project

Region	Population	EUR	AFR	AMR	EAS	SAS
Europe	CEU	**0.4595**	0.0304	**0.2585**	0.0745	**0.1771**
		**0.6691**	0.0027	0.0888	0.0762	**0.1633**
		**0.6858**	0.0062	0.0632	0.0402	**0.2047**
	FIN	**0.4917**	0.0255	**0.2286**	0.0913	**0.1628**
		**0.6816**	0.0018	**0.1338**	0.0703	**0.1125**
		**0.7013**	0.0012	0.0447	0.0885	**0.1642**
	GBR	**0.4617**	0.0302	**0.2588**	0.0742	**0.1751**
		**0.6732**	0.0051	**0.1046**	0.0507	**0.1665**
		**0.7160**	0.0043	0.0460	0.0401	**0.1936**
	IBS	**0.4341**	0.0379	**0.2790**	0.0748	**0.1743**
		**0.6961**	0.0089	0.0882	0.0834	**0.1234**
		**0.7222**	0.0096	0.0576	0.0342	**0.1764**
	TSI	**0.4391**	0.0324	**0.2602**	0.0786	**0.1897**
		**0.6460**	0.0038	0.0878	0.0654	**0.1970**
		**0.6636**	0.0074	0.0560	0.0392	**0.2337**
Africa	ACB	0.0777	**0.7480**	**0.1118**	0.0228	0.0397
		**0.2217**	**0.5736**	0.0588	0.0714	0.0745
		0.0856	**0.8184**	0.0236	0.0114	0.0610
	ASW	**0.1310**	**0.6126**	**0.1559**	0.0398	0.0606
		**0.3291**	**0.4094**	**0.1706**	0.0213	0.0696
		**0.1688**	**0.6343**	0.0946	0.0206	0.0818
	ESN	0.0203	**0.8696**	0.0823	0.0113	0.0165
		0.0010	**0.9863**	0	0.0127	0
		0.0032	**0.9570**	0.0063	0.0016	0.0319
	GWD	0.0362	**0.8216**	**0.1017**	0.0158	0.0246
		0.0581	**0.8657**	0.0119	0.0540	0.0103
		0.033	**0.915**	0.0161	0.0083	0.0277
	LWK	0.0353	**0.8306**	0.0901	0.0178	0.0262
		0.0679	**0.8324**	0.0034	0.0130	0.0833
		0.0092	**0.902**	0.0092	0.0013	0.0778
	MSL	0.0235	**0.8596**	0.0849	0.0131	0.0189
		0.0111	**0.9396**	0.0071	0.0236	0.0186
		0.0178	**0.9111**	0.0377	0.0040	0.0293
	YRI	0.0218	**0.8627**	0.0866	0.0121	0.0168
		0.0122	**0.9639**	0	0.0240	0
		0.0058	**0.9431**	0.0179	0.0031	0.0299
America	CML	**0.2836**	0.0562	**0.4224**	0.0990	**0.1388**
		**0.1937**	0.0007	**0.6988**	0.0435	0.0633
		**0.3298**	0.0182	**0.5242**	0.0376	0.0901
	MXL	**0.2685**	0.0466	**0.4161**	**0.1292**	**0.1395**
		**0.1544**	0.0031	**0.7417**	0.0440	0.0568
		**0.2371**	0.0141	**0.6403**	0.0373	0.0712
	PEL	**0.1516**	0.0301	**0.5517**	**0.1559**	**0.1107**
		0.0388	0	**0.9131**	0.0319	0.0162
		0.0703	0.0068	**0.8640**	0.0346	0.0243

Table S11 – continued from previous page

Region	Population	EUR	AFR	AMR	EAS	SAS
	PUR	**0.2802**	**0.1004**	**0.4070**	0.0815	**0.1310**
		**0.1910**	0.0083	**0.7070**	0.0340	0.0597
		**0.3377**	0.0682	**0.4682**	0.0387	0.0872
East Asia	CDX	0.0836	0.0125	**0.1226**	**0.6509**	**0.1304**
		0.0307	0	**0.1384**	**0.7731**	0.0579
		0.0346	0.0012	**0.1162**	**0.7781**	0.0700
	CHB	0.0918	0.0128	**0.1451**	**0.6170**	**0.1333**
		0.0546	0.0023	**0.1754**	**0.7048**	0.0629
		0.0528	0.0019	**0.1459**	**0.7053**	0.0940
	CHS	0.0863	0.0126	**0.1339**	**0.6353**	**0.1319**
		0.0420	0.0022	**0.1555**	**0.7452**	0.0551
		0.0393	0.0016	**0.1181**	**0.7526**	0.0883
	JPT	0.0889	0.0127	**0.1470**	**0.6215**	**0.1299**
		0.0485	0	**0.2281**	**0.6537**	0.0697
		0.0330	0.0065	**0.1630**	**0.7310**	0.0723
	KHV	0.0869	0.0129	**0.1268**	**0.6345**	**0.1390**
		0.0507	0	**0.1670**	**0.7171**	0.0652
		0.0387	0.0012	**0.1335**	**0.7441**	0.0825
South Asia	BEB	**0.1904**	0.0204	**0.1559**	**0.1854**	**0.4479**
		**0.2241**	0	**0.1405**	**0.2089**	**0.4265**
		**0.2349**	0.0012	0.0974	**0.1861**	**0.4804**
	GIH	**0.2106**	0.0203	**0.1558**	**0.1222**	**0.4910**
		**0.2639**	0.0025	**0.1277**	**0.1263**	**0.4796**
		**0.2562**	0.0012	0.0642	0.0881	**0.5903**
	ITU	**0.1884**	0.0203	**0.1489**	**0.1326**	**0.5097**
		**0.1849**	0	**0.1351**	**0.1390**	**0.5410**
		**0.2211**	0.0012	0.0786	**0.1057**	**0.5934**
	PJL	**0.2207**	0.0215	**0.1631**	**0.1241**	**0.4706**
		**0.2633**	0	**0.1283**	**0.1227**	**0.4857**
		**0.2728**	0.0015	0.0506	0.0813	**0.5937**
	STU	**0.1792**	0.0202	**0.1435**	**0.1361**	**0.5211**
		**0.2193**	0	**0.1148**	**0.1256**	**0.5403**
		**0.2444**	0.0021	0.0605	0.0923	**0.6006**

Bold values are estimates > 0.10

Within each population, top row is estimate using 1KPG as the reference panel.

Middle row is the estimate using the refined panel.

Bottom row is the estimate using the HGDP panel.

Table S12: Run time and memory usage comparison on 1000 Genomes Project data

Metrics	FRAME	iAdmix	ADMIXTURE	FLARE	RFMix	Gnomix	Recomb-Mix
CPU (hrs)	0.98	4.25	17.37	19	952.86	1201.17	15.64
Wallclock (hrs)	1.01	4.56	17.73	0.42	10.26	9.68	15.88
Peak Memory(in GB)	0.43	5.70	40.07	64	82.35	38.94	26.21

Table S13: Commands for all the tools benchmarked

Tools	Commands
FRAME	`python run.py -i ./vcfs/-l 500 -r ref.pop -s query_samples -o ./out/ frame -n sitesPerChrom -c 20`
FLARE	`java -Xmx20g -jar flare.jar ref=20.ref.vcf gt=20.query.vcf map=20.flare.genmap ref-panel=ref.pop probs=false out=flare.estimate`
FLARE+	`java -Xmx20g -jar flare.jar ref=20.ref.vcf gt=20.query.vcf map=20.flare.genmap ref-panel=ref.pop probs=true out=flare.probs.estimate`
ADMIXTURE	`./admixture --cv --supervised chr20.query.plink.fixed.bed 3`
iAdmix	`python2 runancestry.py --freq ./iadmix/chr20.ref.af --plink ./ plinkinputs/chr20.query --path ./iAdmix/ancestry --strand 1 --out ./out/iadmix/chr20.out`
RFMix	`rfmix -f 20.query.vcf -r 20.ref.vcf -m ref.pop -g chr20.decodeMap.txt -o ./out/rfmix --chromosome=20`
Gnomix	`python3 gnomix.py 20.query.vcf ./out/gnomix 20 False chr20.decodeMap.txt 20.ref.vcf ref.pop`
Gnomix+	`python3 gnomix.py 20.query.vcf ./out/gnomix 20 False chr20.decodeMap.txt 20.ref.vcf ref.pop`
Recomb-Mix	`RecombMix_v0.6 -p 20.ref.vcf -q 20.query.vcf -a ref.pop -o ./out/ recomb_mix -i inferred_ancestral_values.txt -g chr20.decodeMap.txt`
Recomb-Mix-f	`RecombMix_v0.6 -p 20.ref.vcf -q 20.query.vcf -a ref.pop -f 0.1 -o ./ out/recomb_mix -i inferred_ancestral_values.txt -g chr20.decodeMap.txt`
